# Engineering of *Corynebacterium glutamicum* for the synthesis of aromatic compounds

**DOI:** 10.1007/s00253-025-13520-3

**Published:** 2025-05-30

**Authors:** Jan Marienhagen

**Affiliations:** 1 Institute of Bio- and Geosciences, Forschungszentrum Jülich, IBG-1: Biotechnology, 52425 Jülich, Germany; 2 Institute of Biotechnology, RWTH Aachen University, Worringer Weg 3, 52074 Aachen, Germany

**Keywords:** *Corynebacterium glutamicum*, Metabolic engineering, Aromatic, Aromatic compound, Shikimate pathway, Plant polyphenol, Polyketide

## Abstract

**Abstract:**

A significant proportion of industrially important small molecules are aromatic, and the majority of these compounds are produced chemically, relying heavily on fossil resources. In bacteria and plants, the shikimate pathway and related biosynthetic routes serve as the primary sources of aromatic compounds. Microbial cell factories, which are poised to play a central role in the emerging bio-based economy, provide a sustainable alternative for producing commercially valuable aromatics from renewable resources. *Corynebacterium glutamicum*, already established as an industrial workhorse for the large-scale production of various amino acids, can be engineered to overproduce aromatic compounds derived from the shikimate pathway. Furthermore, the functional integration of heterologous or synthetic pathways enables access to high-value natural products, such as plant polyphenols and other polyketides. This review highlights recent advancements in the metabolic engineering of *C. glutamicum* for the sustainable production of these aromatic compounds.

**Key points:**

•* C. glutamicum’s high tolerance to aromatic compounds is key to aromatics production.*

•* Detailed physiological insights enable access to shikimate pathway-derived products.*

• *Diverse plant (poly)phenols and other aromatic polyketides can be produced.*

**Graphical Abstract:**

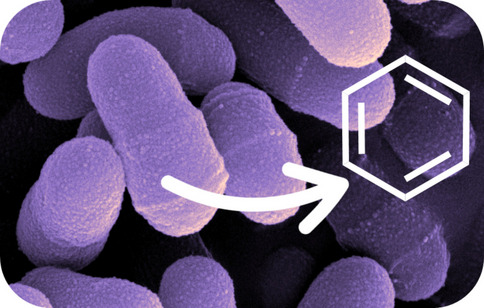

## Introduction

Aromatic compounds constitute a diverse class of chemicals with numerous industrial applications, including their use as organic solvents, dyes, fuels, food, and feed additives, as well as building blocks for pharmaceuticals and polymer materials (Noda and Kondo 2017; Averesch and Krömer 2018). Currently, the vast majority of aromatic chemicals are produced through the chemical conversion of BTX (benzene, toluene, and xylene) derived from petroleum-based feedstocks or natural gas (Krömer et al. 2013). However, growing concerns over the consumption of fossil resources and the environmental impacts of chemical production, in particular significant CO_2_ emissions, have spurred efforts to develop more sustainable production methods in recent years (Dickey et al. 2021). Alternatively, catalytic fast pyrolysis of biomass feedstocks (e.g., lignin) to bio-oil offers a potential pathway for producing commercially interesting aromatics, but faces challenges such as low catalyst stability, high-energy requirements, and the accumulation of undesired by-products (Yildiz et al. 2016; Nekhaev and Maksimov 2021).

In comparison, microbial production of aromatic compounds represents a promising alternative with several advantages. Typically, microbial production from abundant and inexpensive sugar feedstocks is more environmentally friendly compared to chemical synthesis, since it avoids the use of heavy metals, organic solvents, and strong acids or bases. Additionally, microorganisms exhibit rapid growth, enabling short production times, and microbial fermentation processes are scalable—from laboratory bench-top experiments to industrial-scale fermenters with capacities of several hundred cubic meters (Nielsen et al. 2022). Most bacteria and archaea, but also protozoa, fungi, algae, and plants (but notably not animals!) possess the metabolic capacity to synthesize various aromatic compounds via the shikimate pathway, a source of both, aromatic and nonaromatic compounds with commercial value (Herrmann and Weaver 1999; Shende et al. 2024). To harness the potential of the shikimate pathway for producing valuable small aromatic molecules, organisms such as *Escherichia coli* and *Saccharomyces cerevisiae* have been studied for many years (Jiang and Zhang 2016; Liu et al. 2020).

Another microorganism considered to be well suited for the production of aromatic compounds is *Corynebacterium glutamicum*, a Gram-positive, non-motile and non-pathogenic soil bacterium. This organism has been adopted industrially for the production of proteinogenic amino acids since its discovery as l-glutamate-overproducing microorganism in Japan more than 70 years ago (Kinoshita et al. 1957). *C. glutamicum* demonstrates several physiological properties advantageous to fermentative microbial production, such as (i) high sugar consumption rates under either aerobic or anaerobic conditions, regardless of cell density, (ii) a strong tolerance to osmotic stress, and (iii) the capability of simultaneously utilizing sugar mixtures without carbon catabolite repression (Kogure and Inui 2018; Zha et al. 2023). Extensive scientific work in laboratories worldwide has provided a comprehensive physiological and genetic understanding of this organism. This work facilitated the development of detailed genome-scale models and advanced -*omics* tools for its global analysis (Parise et al. 2020; Gong et al. 2024). In addition, numerous methods for the genetic manipulation of *C. glutamicum* are available, enabling the metabolic engineering of this bacterium for diverse applications (Nešvera and Pátek 2011; Jiang et al. 2017). Hence, it is no surprise that these extensive research efforts turned *C. glutamicum* into a versatile microbial platform organism for the synthesis of approximately 100 small molecules of biotechnological interest. These compounds include alcohols, organic acids, amino acid derivatives, diamines, fatty acids, and terpenoids (Wolf et al. 2021; Zha et al. 2023). In this context, it is astonishing that the potential of *C. glutamicum* for producing aromatic compounds beyond aromatic amino acids is relatively underutilized. The described high resistance to increased concentrations of cytotoxic aromatic compounds such as hydroxybenzoic acids or phenylpropanoids would be a significant advantage of *C. glutamicum*–based cell factories for aromatics production (Kallscheuer et al. 2016a; Kitade et al. 2018). For instance, in direct comparison to *Pseudomonas putida*, another robust biotechnological workhorse, *C. glutamicum* grows faster and reaches a higher biomass yield when cultivated in the presence of 10 g L^−1^ anthranilate (Kuepper et al. 2020). This tolerance of *C. glutamicum* is partly attributed to the characteristic outer membrane rich in mycolic acids, which acts as a permeability barrier (Marchand et al. 2012). Additionally, the extensive catabolic network for the degradation of aromatic compounds in *C. glutamicum* is well understood, and can easily be manipulated to enable the accumulation of valuable pathway intermediates or to prevent the degradation of precursor molecules or target compounds (Kallscheuer et al. 2016a).

In this review, I summarize advances in metabolic engineering of *C. glutamicum* for the synthesis of aromatics over the past 10 years, covering shikimate pathway–derived compounds, plant (poly)phenols, and other polyketides.

## Production of shikimate pathway intermediates and aromatic amino acids

**Fig. 1:**
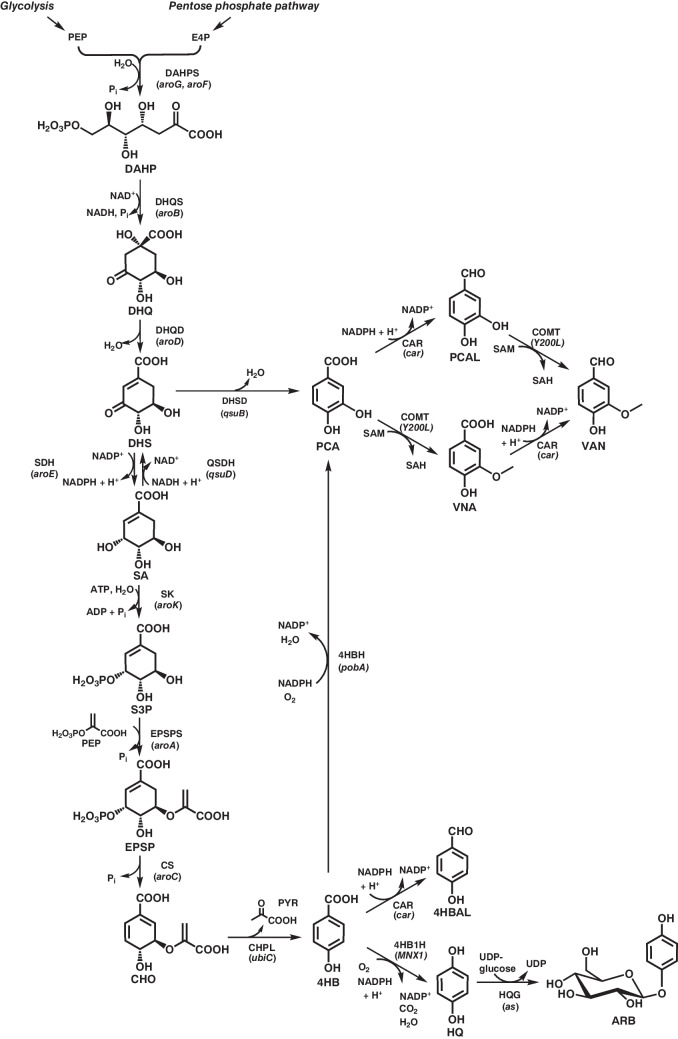
Schematic overview of the shikimate pathway in *C. glutamicum* and relevant metabolic pathways to biotechnologically interesting aromatic compounds derived thereof. Compound names and (heterologous) enzymes are indicated. Genes for the encoding enzymes are given in brackets. Precursors, co-substrates, intermediates, and products (for the sake of consistency, all carboxyl groups are depicted in their protonated state): 4HB, 4-hydroxybenzoate; 4HBAL, 4-hydroxybenzaldehyde; ARB, β-arbutin; CHO, chorismate; DAHP, 3-deoxy-d-arabinoheptulosonate-7-phosphate; DHQ, 3-dehydroquinate; DHS, 3-dehydroshikimate; E4P, erythrose 4-phosphate; EPSP, 5-enolpyruvyl-shikimate-3-phosphate; HQ, hydroquinone; PCA, protocatechuate; PCAL, protocatechuic aldehyde; PEP, phosphoenolpyruvate; PYR, pyruvate; S3P, shikimate-3-phosphate; SA, shikimate; SAH, *S*-adenosylhomocysteine; SAM, *S*-adenosylmethionine; UDP, uracil-diphosphate; UDP-glucose, uracil-diphosphate glucose; VAN, vanillin; VNA, vanillate. Enzymes (genes): 4HBH (*pobA*), 4-hydroxybenzoate-3-hydroxylase; 4HB1H (*MNX1*), 4-hydroxybenzoate-1-hydroxylase (*Candida parapsilosis* CBS604); CAR, (*car*) carboxylic acid reductase (e.g., *Nocardia iowensis*); CHPL (*ubiC*), chorismate-pyruvate lyase (*E. coli*); CS (*aroC*), chorismate synthase; COMT (*y200L*), catechol-*O*-methyltransferase (*Rattus norvegicus*); DAHPS (*aroG*, *aroF*), 3-deoxy-arabinoheptulosonate-7-phosphate synthase; DHQD (*aroD*), 3-dehydroquinate dehydratase; DHQS (*aroB*), 3-dehydroquinate synthase; DHSD (*qsuB*), dehydroshikimate dehydratase; EPSPS (*aroA*), 5-enolpyruvyl-shikimate-3-phosphate synthase; HQG (*as*), hydroquinone UDP-dependent glycosyltransferase (β-arbutin synthase, *Rauvolfia serpentin*); QSDH (*qsuD*), quinate/shikimate dehydrogenase; SDH (*aroE*), shikimate dehydrogenase; SK (*aroK*), shikimate kinase

**Fig. 2:**
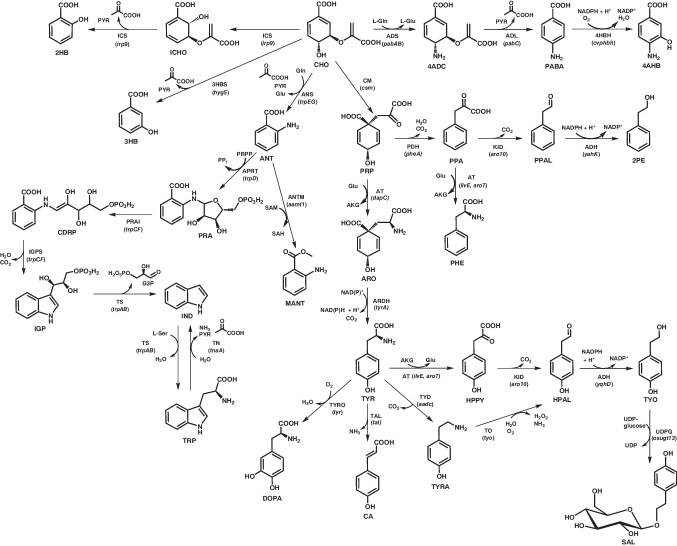
Schematic overview of metabolic strategies starting from chorismate, which are leading to the three aromatic amino acids and other compounds of biotechnological interest in *C. glutamicum*. Precursors, co-substrates, intermediates and products (for the sake of consistency, all carboxyl groups are depicted in their protonated state): 2HB, 2-hydroxybenzoate (salicylate); 2PE, 2-phenylethanol; 3HB, 3-hydroxybenzoate; 4 AHB, 4-amino-3-hydroxybenzoate; 4 ADC, 4-amino-4-deoxychorismate; AKG, α-ketoglutarate; ANT, anthranilate; ARO, arogenate (pretyrosine); CA, *para*-coumarate (4-hydroxycinnamic acid); CDRP, 1-(2-carboxyphenylamino)−1-deoxy-d-ribulose-5-phosphate; CHO, chorismate; DOPA, 3,4-dihydroxyphenyl-l-alanine; G3P, glyceraldehyde-3-phosphate; HPAL, hydroxyphenylacetaldehyde; HPPY, hydroxyphenylpyruvate; ICHO, isochorismate; IGP, indole-3-glycerol phosphate; IND, indole; MANT, methylanthranilate; PABA, *para*-aminobenzoate; PHE, l-phenylalanine; PPA, phenylpyruvate; PRA, *N*-(5'-phosphoribosyl) anthranilate; PPAL, phenylacetaldehyde; PPRP, phosphoribosylpyrophosphate; PRP, prephenate; PYR, pyruvate; SAH, *S*-adenosyl-l-homocysteine; SAL, salidroside; SAM, *S*-adenosyl-l-methionine; TRP, l-tryptophan; TYO, tyrosol; TYR, l-tyrosine; TYRA, tyramine; UDP, uracil-diphosphate; UDP-glucose, uracil-diphosphate glucose. Enzymes (*genes*): 3HBS (*hyg5*), chorismatase (3-hydroxybenzoate synthase, *Streptomyces hygroscopicus*); 4HBH (*cvphbh*), 4-hydroxybenzoate-3-hydroxylase (e.g., *Caulobacter vibrioides*); ADH (*yahK, yqhD*), alcohol dehydrogenase (*E. coli*); ADL (*pabC*), 4-amino-4-deoxychorismate lyase (e.g., *Xenorhabdus bovienii*); ADS (*pabAB*), 4-amino-4-deoxychorismate synthase component I and I (e.g., *Corynebacterium callunae*); ANS (*trpEG*), anthranilate synthase; ANTM (*aamt1*), anthranilate methyltransferase (*Zea mays*); APRT (*trpD*), anthranilate phosphoribosyltransferase; ARDH (*tyrA*), arogenate dehydrogenase; AT (*dapC, ilvE, aroT*), aminotransferase; CM (*csm*), chorismate mutase; ICS (*irp9*), isochorismate synthase/isochorismate pyruvate lyase (salicylate synthase, *Yersinia enterocolitica*); IGPS (*trpCF*), indole-3-glycerol phosphate synthase; UDPG (*osugt13*), UDP-dependent glycosyltransferase (*Oryza sativa*); PDH (*pheA*), prephenate dehydratase; PRAI (*trpCF*), phosphoribosylanthranilate isomerase; KID (*aro10*), 2-ketoisovalerate decarboxylase (*Saccharomyces cerevisiae*); TAL (*tal*), phenylalanine/tyrosine ammonia lyase (*Flavobacterium johnsoniae*); TN (*tnaA*), tryptophanase (e.g., *Providencia rettgeri*); TO (*tyo*) tyramine oxidase (*Kocuria rhizophila*); TS (*trpAB*), tryptophan synthase; TYD (*aadc*), tyrosine decarboxylase (e.g., *Levilactobacillus brevis*); TYRO (*tyr*), tyrosinase (*Ralstonia solanacearum*)

The canonical shikimate pathway, which also represents the primary route for the biosynthesis of aromatic compounds in *C. glutamicum*, begins with an enzyme-catalyzed aldol-like condensation of phosphoenolpyruvate (PEP), derived from glycolysis, and d-erythrose-4-phosphate (E4P), originating from the pentose phosphate pathway (Fig. 1). This decisive reaction, yielding 3-deoxy-d-arabino-heptulosonate-7-phosphate (DAHP), is catalyzed by two feedback-regulated DAHP-synthase isoenzymes (AroF, AroG). The pathway then proceeds through a series of six additional enzymatic steps, culminating in the formation of chorismate (CHO), a central precursor for the synthesis of the three aromatic amino acids l-phenylalanine (PHE), l-tryptophan (TRP), and l-tyrosine (TYR) (Fig. 2) as well as folate (vitamin B_9_) and ubiquinone-10 (coenzyme Q10).

The eponymous and nonaromatic intermediate in the pathway is shikimate (SA), which has commercial value. SA has attracted significant interest as a pharmaceutical building block, most notably as precursor for the industrial synthesis of the anti-influenza drug Tamiflu (oseltamivir phosphate) (Jackson et al. 2011). In 2015, a *C. glutamicum* variant capable of accumulating up to 11.3 g L^−1^ of SA in fed-batch cultivations was reported (Zhang et al. 2015). To construct this strain, modularized gene libraries encoding the first four enzymes of the shikimate pathway were screened to identify the best SA-accumulating variants. These enzymes include DAHP synthase (DAHPS, encoded by *aroG*), 3-dehydroquinate synthase (DHQS, encoded by *aroB*), 3-dehydroquinate dehydratase (DHQD, encoded by *aroD*), and SA dehydrogenase (SDH, encoded by *aroE*) (Fig. 1). The best-performing enzyme combination was then implemented in a *C. glutamicum* strain with deletion of the *aroK*-gene. This gene encodes the essential shikimate kinase (SK), which phosphorylates SA to shikimate-3-phosphate (S3P) (Fig. 1). As a result, the SA-producer was auxotrophic for all three aromatic amino acids and *para*-aminobenzoate (PABA) requiring supplementation with complex medium components for growth and SA production. In another study, a *C. glutamicum* R variant capable of utilizing xylose and arabinose in combination with glucose was reengineered to accumulate SA (Kogure et al. 2016). This strain was also devoid of any SK activity, rendering it auxotrophic for PHE, TRP, TYR, and PABA, and also carried a plasmid for the episomal expression of *aroGBDE*. Furthermore, two other genes (*qsuB*, dehydroshikimate dehydratase (DHSD) and *qsuD*, quinate/SA dehydrogenase (QSDH) (Fig. 1) involved in the consumption of SA and its precursor molecules 3-dehydroshikimate (DHS) and 3-dehydroquinate (DHQ) were disrupted. Additional modifications of glucose uptake (elimination of the phosphotransferase (PTS) system) and its conversion via the glycolysis to improve PEP availability allowed for the construction of the final variant capable of accumulating up to 141 g L^−1^ SA from glucose as sole carbon and energy source in high-density resting cell fermentations. Under similar conditions, 137 g L^−1^ SA could be obtained when a mixture of glucose, xylose, and arabinose was used as substrate. Notably, several shikimate pathway intermediates, particularly DHS, accumulated at gram-scale levels. More recently, Sato and coworkers constructed a genetically similar *C. glutamicum* variant for SA production, maintaining an unmodified phosphotransferase system while integrating additional chromosomal copies of selected genes involved in the shikimate pathway (*aroGBE*) (Sato et al. 2020). In complex medium supplemented with the three essential aromatic amino acids and PABA, the strain accumulated 13.1 g L^−1^ SA from glucose under batch conditions. Additionally, the strain was engineered to utilize cellobiose by expressing a β-glucosidase gene and secretion of this corresponding enzyme. Under the same cultivation conditions, SA accumulation reached 13.8 g L^−1^, with yield comparable to that obtained from glucose.

Anthranilate (ANT) is not an intermediate of the shikimate pathway, but is directly synthesized from CHO by ANT synthase (ANS), and represents the first intermediate in the biosynthetic route to TRP (Fig. 2). ANT can be used for the production of polyurethanes and as precursor for various food additives and dyes (Wiklund and Bergman 2006). To construct a *C. glutamicum*–based ANT producer, the *trpD* gene, encoding the anthranilate phosphoribosyltransferase (APRT), was deleted (Luo et al. 2019). As a result, the modified variant became TRP-auxotrophic, since APRT catalyzes the essential step converting ANT to *N*-(5'-phosphoribosyl) anthranilate (PRA) during TRP biosynthesis. Similar to the strategy employed for constructing SA-producing *C. glutamicum* variants, *qsuB* and *qsuD* were deleted to eliminate competing pathways for protocatechuate and quinate synthesis, respectively. Additionally, promoter replacement was performed to enhance the expression of *aroB* and *aroK*. Further optimization included episomal expression of a feedback-resistant gene for an AroG variant, along with minor modifications in sugar metabolism. These metabolic engineering strategies enabled the strain to accumulate 26.4 g L^−1^ ANT during fed-batch cultivation in defined medium with TRP supplementation. More recently, a prototrophic *C. glutamicum* variant for ANT production was constructed, which eliminated the need for TRP supplementation. This strain produced 5.9 g L^−1^ ANT in fed-batch bioreactor cultivations, using a glucose-xylose mixture as carbon source (Mutz et al. 2024). Similar to the approach by Luo et al., this strain carries a plasmid encoding a feedback-resistant DAHPS to enhance the flux into the shikimate pathway. Additional modifications included modulation of the translation efficiency of *aroK* (to increase overall SK activity) and *trpD* (to reduce APRT activity) by start codon replacements, thereby minimizing ANT conversion toward TRP biosynthesis. However, the most significant improvement in ANT production was achieved by introducing an engineered ANS variant that is unresponsive to feedback inhibition by TRP. To develop this optimized enzyme, component I of ANS (TrpE) was engineered using a biosensor-guided in vivo screening strategy to identify suitable variants (Flachbart et al. 2021). In a separate study, adaptive laboratory evolution (ALE) was employed to enhance *C. glutamicum*’s tolerance to ANT. Through sequential batch cultivations in shake flasks in the presence of ANT concentrations up to 25 g L^−1^, a variant with improved tolerance was obtained (Kuepper et al. 2020). In comparison to the *C. glutamicum* ATCC 13032 wild type, the best-performing evolved variant exhibited a 2.2-fold increased growth rate and a 1.4-fold higher final optical density of the culture. However, the specific mutations responsible for enhanced ANT tolerance remain to be identified.

The microbial production of the three aromatic amino acids using *C. glutamicum* has a long-standing history (Ikeda 2006). However, over the past decade, only a few studies have focused on engineering this bacterium to enhance the production. PHE is produced for medical-, pharmaceutical-, feed-, and nutritional applications. In particular for the synthesis of aspartame, an artificial, non-saccharide sweetener used in foods and beverages PHE is required (Bang et al. 2021). With the aim of constructing a PHE-overproducing strain, Zhang and colleagues overexpressed genes from the shikimate pathway and central sugar metabolism in a variant that already carried endogenous but engineered genes encoding a feedback-resistant DAHPS (AroF) and the prephenate dehydratase (PDH, PheA), both key enzymes in PHE biosynthesis (Fig. 2) (Zhang et al. 2015). Subsequently, the accumulation of PHE and SA was evaluated to identify additional targets for metabolic engineering. Combinatorial expression of identified key genes revealed that moderate expression of genes involved in conversions upstream of SA, combined with stronger expression of genes downstream of SA, was most effective for maximizing PHE synthesis while minimizing SA accumulation. Further modifications including improved sugar uptake, reduced PHE (re)uptake (by inactivating the gene for the transporter AroP), and elimination of acetate and lactate formation, contributed to the engineering of the best-performing variant. This strain accumulated 15.6 g L^−1^ PHE in fed-batch fermentations containing glucose and corn steep liquor. Interestingly, a PHE-antimetabolite screening using 4-fluorophenylalanine, published nine years later, identified mutations in three of the previously identified key target genes contributing to PHE overproduction: *aroG* (isoenzyme of *aroF*), *pheA* and *aroP* (Tachikawa et al. 2024). A *C. glutamicum* variant engineered for episomal overexpression of the mutated *aroG* and *pheA* variants and with a chromosomal deletion of *aroP* allowed for the production of up to 6.11 g L^−1^ PHE in shake flask cultivations.

TRP is a nutritional supplement, has several medical applications, and is added to poultry and livestock feed to enhance growth rates, reproduction, and overall animal health (Ikeda 2006). For the production of TRP using *C. glutamicum*, a strain devoid of any chorismate mutase (CM) activity was constructed by deleting the essential CM-encoding *csm* gene in a strain background with various chromosomal modifications of genes involved in the shikimate pathway, leading to PHE- and TYR-auxotrophy (Fig. 2) (Mindt et al. 2023). To alleviate attenuation control of the endogenous *trp* operon, which encodes all enzymes of the biosynthetic pathway from CHO to TRP, the gene for the leader peptide *trpL* was also deleted. In addition, the strain was engineered through chromosomal expression of a mutated, endogenous *trpE* gene encoding a TRP-insensitive ANS variant. This was complemented by episomal heterologous expression of an additional TRP-insensitive *trpE* copy from *E. coli*, along with the *trpD* gene for the other ANS subunit in the same bacterium. The resulting strain accumulated 2.11 g L^−1^ TRP in shake flask cultivations.

Metabolic engineering at the pathway branch points of CHO and prephenate (PRP) were also the first step towards constructing a *C. glutamicum* production strain for TYR (Fig. 2) (Kurpejović et al. 2023). TYR is utilized in the biosynthesis of catecholamine neurotransmitters (e.g., dopamine, norepinephrine, and epinephrine), hormones, and melanin, with applications in neuropharmacology, metabolic disorder treatments, and dietary supplementation (Lütke-Eversloh et al. 2007). To achieve TYR production, the start codons of the coding sequences of *trpE*, *pheA*, and *pat* (also known as *aroT*, encoding an aminotransferase) were altered from the canonical ATG to the less preferred TTG in the chromosome, aiming to redirect pathway flux to TYR biosynthesis. Evaluation of the resulting variants revealed that the start codon modification of *pheA* had the most pronounced effect on TYR-production. However, this came at the cost of PHE bradytrophy, which could be mitigated by supplementing 0.5 mM PHE. Unlike some of the aforementioned studies, neither deletion of *qsuABD* nor elimination of PTS-mediated glucose uptake had a positive effect on product formation in this strain background. Nonetheless, the functional implementation of the isomerase pathway, achieved through the overexpression of genes encoding a xylose isomerase and a xylulokinase, enabled the co-utilization of xylose as carbon and energy source. The highest TYR concentrations obtained during batch cultivations in shake flasks were 3.2 g L^−1^ on glucose and 3.6 g L^−1^ on a 1:3 (w/v) glucose-xylose mixture.

## Products derived of the shikimate pathway

Protocatechuate (PCA) is a naturally occurring aromatic acid with notable properties including antioxidant, antiviral and anticancer activity effects against various human cancer cell lines (Song et al. 2020). PCA can be synthesized through two metabolic routes (1) dehydration of the shikimate pathway intermediate DHS, catalyzed by the DHSD, or (2) hydroxylation of 4-hydroxybenzoate (4HB) by the 4-hydroxybenzoate-3-hydroxylase (4HBH) (Fig. 1). The latter precursor, 4HB, can be directly derived from CHO through the activity of a chorismate lyase (CHPL). Okai and colleagues opted for the latter metabolic strategy utilizing the CHPL UbiC from *E. coli* (Okai et al. 2016). The gene encoding this enzyme was heterologously expressed in an undisclosed PHE-producing *C. glutamicum* F (ATCC 21420) variant, enabling the production of 1.14 g L^−1^ PCA from glucose during fed-batch bioreactor cultivations. Two years later, a *C. glutamicum* strain was engineered to produce PCA via DHSD-mediated dehydration of DHS (Kallscheuer and Marienhagen 2018). To prevent PCA degradation, the *C. glutamicum* DelAro^5^ variant lacking the β-ketoadipate pathway, the gentisate pathway, and other catabolic routes for aromatics to prevent PCA degradation was used. In combination with reduced citrate synthase activity for improved precursor availability and heterologous expression of a feedback-insensitive DAHPS from *E. coli*, this *C. glutamicum* strain accumulated up to 2.0 g L^−1^ PCA from glucose in shake flask cultivations. In a subsequent study, this strain was equipped with a plasmid for the episomal expression of genes encoding xylose isomerase and xylulokinase facilitating xylose utilization to enhance E4P availability (Labib et al. 2021). Additionally, the endogenous *pyk* gene encoding pyruvate kinase was deleted in this strain to increase the intracellular PEP pool, enabling growth-decoupled PCA synthesis when xylose was the sole carbon source. Under growth-decoupled bioreactor conditions, PCA accumulation reached 9.5 g L^−1^, when glucose and xylose were used as orthogonal carbon substrates for biocatalyst provision and product synthesis, respectively. Kogure and coworkers demonstrated that both aforementioned routes to PCA can be utilized simultaneously to enhance product formation (Kogure et al. 2021). With the best strain, concentrations of up to 82.7 g L^−1^ PCA were achieved with growth-arrested cells cultured at high densities.

Notably, PCA also serves as precursor for pseudoaromatic dicarboxylic acids, such as 2-pyrone-4,6-dicarboxylic acid or 2,4-pyridine dicarboxylic acid. Since these compounds are not true aromatics, they are not discussed here. Nonetheless, it is worth mentioning that *C. glutamicum* has recently been engineered to produce such compounds at gram scale (Cho et al. 2024).

The previously mentioned 4HB is not only a direct precursor of PCA; it is also a product of biotechnological interest, similar to other monohydroxylated benzoic acids such as 2-hydroxybenzoate (2HB) and 3-hydroxybenzoate (3HB). While 3HB and 4HB serve as polymer building blocks, 2HB—also known as salicylic acid—is the precursor of acetylsalicylic acid, better known as the painkiller aspirin (del Olmo et al. 2017). In the same study investigating the aforementioned PCA production from DHS, it could be demonstrated that 2HB, 3HB and 4HB can be synthesized from CHO (Fig. 1, Fig. 2) (Kallscheuer and Marienhagen 2018). The *C. glutamicum* DelAro^5^-strain lacking the catabolic network for aromatics degradation was successfully converted into a 2HB producer. Upon functional expression of the *irp9* gene—encoding a bifunctional isochorismate synthase/isochorismate pyruvate lyase (ICS, salicylate synthase) from *Yersinia enterocolitica*—this strain synthesized up to 0.01 g L^−1^ 2HB (Fig. 2). *Streptomyces hygroscopicus* is known to possess the 3-hydroxybenzoate synthase (3HBS, chorismatase) Hyg5, which catalyzes hydrolysis and concomitant dehydration of CHO leading to 3HB. Up to 0.03 g L^−1^ 3HB could be synthesized by *C. glutamicum* DelAro^5^ upon functional episomal implementation of a codon-optimized *hyg5* gene (Fig. 2). For 4HB production, CHPL UbiC from *E. coli* was used, allowing for a final product titer of 3.3 g L^−1^ 4HB (Fig. 1) (Kallscheuer and Marienhagen 2018). In the same year, a very similar *C. glutamicum* strain for 4HB was reported (Syukur Purwanto et al. 2018). This strain was auxotrophic for all three aromatic amino acids due to deletions of *trpE* and *csm*, essential genes encoding the ANS subunit TrpE and CSM. Combined with expression of a gene for a CHPL with reduced sensitivity to product inhibition, and extensive modifications of the shikimate pathway the resulting variant produced up to 19 g L^−1^ 4HB in fed-batch fermentations, using complex medium with the essential supplementation of PHE, TRP and TYR. Simultaneously, similar efforts by Kitade and colleagues culminated in a highly engineered *C. glutamicum* R variant expressing a highly active *ubiC* gene from *Providencia rustigianii*. This strain accumulated up to 36.6 g L^−1^ 4HB in jar fermenters using aerobic growth–arrested cells (Kitade et al. 2018).

Aldehydes derived from various mono- and dihydroxybenzoates serve as flavors, fragrances or as pharmaceutical precursors with vanillin being a prominent example for such compounds (Fig. 1) (Kunjapur and Prather 2015). However, the production of aromatic aldehydes is challenging due to their rapid reduction to the corresponding alcohols. To address this challenge, a comprehensive screening of 27 candidate proteins for aromatic aldehyde reductase activity was conducted in a *C. glutamicum* strain engineered for the production of 4-hydroxybenzaldehyde (4HBAL) (Kim et al. 2022). This screening identified the gene NCgl0324 to encode an enzyme with the undesired reductase activity. Deletion of the corresponding gene led to the accumulation of 1.36 g L^−1^ 4HBAL. Further experiments demonstrated that this deletion also enhanced the synthesis of protocatechuate aldehyde (PCAL) and vanillin (VAN, 4-hydroxy-3-methoxybenzaldehyde) in shake flask cultures. Deletion of the very same gene also proved crucial for developing a process for the biotransformation of vanilliate (VNA) into VAN (Matsuzawa et al. 2024). In this context, a screening of 17 carboxylic acid reductase (CAR) candidate proteins identified three enzymes capable of reducing of up to 21 g L^−1^ VNA to VAN in jar fermenters.

CHO not only serves as a precursor for the three aromatic amino acids and various mono- and dihydroxylated benzoates and their derivatives, but also for PABA mentioned before. PABA is an intermediate in endogenous folate synthesis and an important building block for commercial drugs and azo dyes (Kluczyk et al. 2012). For PABA production in *C. glutamicum* R, three heterologous genes encoding 4-amino-4-deoxychorismate synthase components I and II (ADS, encoded by *pabAB* from *Corynebacterium callunae*) and 4-amino-4-deoxychorismate lyase (ADL, encoded by *pabC* from *Xenorhabdus bovienii*) were episomally expressed (Fig. 2) (Kubota et al. 2016). However, PABA accumulation was accompanied by a glycation byproduct, likely formed through non-enzymatic reaction of PABA’s primary amino group with glucose’s aldehyde group. Acidification effectively decomposed this byproduct back into PABA and glucose. The best strain yielded up to 43 g L^−1^ PAPA in fed-batch fermentations. PABA can be further hydroxylated to 4-amino-3-hydroxybenzoate (4 AHB), a building block for polybenzoxazole polymers—high-performance materials with exceptional mechanical strength and thermal stability (Fig. 2) (Hong et al. 2003). Nonaka and colleagues built on the strain engineered for PABA production and screened six 4-hydroxybenzoate-3-hydroxylases (4HBH) for their ability to hydroxylate PABA (Nonaka et al. 2023). *Cv*PHBH from *Caulobacter vibrioides* emerged as the most promising candidate and was further adapted for applications in *C. glutamicum* through protein engineering. In fed-batch fermentations using complex medium with glucose as carbon and energy source, the best variant accumulated up to 13.5 g L^−1^ 4 AHB.

Methylanthranilate (MANT) is widely used to impart a grape scent and flavor in food and cosmetics industries, but is currently produced via petroleum-based processes. To develop a more sustainable alternative, Luo and colleagues modified their previously engineered TRP-auxotrophic *C. glutamicum* strain for ANT production by introducing a gene encoding anthranilic acid methyltransferase (ANTM) from *Zea mays* (Fig. 2) (Luo et al. 2019). During *O*-methylation of ANT, this enzyme consumes *S*-adenosyl-l-methionine (SAM) as methyl donor prompting further strain engineering to enhance intracellular SAM availability. This was achieved by recycling *S*-adenosyl-l-homocysteine (SAH), the demethylation product of SAM through episomal overexpression of the endogenous gene encoding the SAH hydrolase. In biphasic fed-batch cultures using a defined medium containing glucose and TRP, the best variant enabled the production of 5.74 g L^−1^ MANT.

Indole (IND), the direct precursor of TRP, is a key signaling molecule in bacteria and plants and is also prized in the food and fragrance industries for its jasmine-like aroma (Ferrer et al. 2023). Additionally, halogenated and oxygenated IND derivatives can serve as colorants, and hold promise for therapeutic applications in treating human diseases. In nature, IND is synthesized either from indole-3-glycerol phosphate (IGP), an intermediate in the TRP biosynthesis pathway, or from TRP via bacterial tryptophanases (TNs) (Fig. 2). Both metabolic options were implemented in *C. glutamicum*, enabling the microbial production of this aromatic heterocycle: In 2022, Ferrer and colleagues leveraged the IGP lyase activity of the α-subunit of the endogenous bifunctional tryptophan synthase (TS) to produce IND in strains supplying IGP (Fig. 2) (Ferrer et al. 2022). In the context of this study, plant-derived “stand-alone” enzymes possessing only the IGP lyase activity were screened for potential application in *C. glutamicum*. Among six plant-derived enzymes tested, the IGP lyase from common wheat (*Triticum aestivum*) exhibited a performance comparable to that of the endogenous TS α-subunit. Increased IND production was achieved by deleting the *csm* gene, which rendered the resulting variant auxotrophic for TYR and PHE, while rerouting the metabolic flux in the direction of ANT. However, other modifications were required to enable higher IND titers. These modifications included the deletion of the *trpL* leader peptide gene and the expression of a TRP-insensitive ANS variant-encoding gene from *E. coli*. Ultimately, with application of in situ product recovery (ISPR), using tributyrin as a second phase, the best strains produced up to 0.67 g L^−1^ IND. In the same year, the same group established IND production from supplemented TRP in whole-cell biotransformations using a *C. glutamicum* strain with heterologous expression of the TN gene from the gamma-proteobacterium *Providencia rettgeri* (Mindt et al. 2022). The highest IND production was achieved with a strain co-expressing the native aromatic amino acid permease gene *aroP* to enhance TRP uptake. Product toxicity was mitigated through ISPR, utilizing dibutyl sebacate as second organic phase. The approach enabled complete TRP conversion with an IND product titer of 5.7 g L^−1^. One year later, the concept of TN-based IND production was expanded to allow de novo indole production from glucose, eliminating the need for TRP supplementation (Mindt et al. 2023). Initially, the *csm* gene was deleted in an existing SA-accumulating *C. glutamicum* starting strain to increase CHO availability, although this rendered the strain auxotrophic for PHE and TYR. Upon introduction of plasmids for the heterologous expression of the ANS encoding gene from *E. coli* (Ferrer et al. 2022) TRP accumulation was observed. Combined with other genetic modifications and ISPR using tributyrin, a final IND titer of 1.38 g L^−1^ was achieved. Notably, decoupling biomass production from IND production by aerobic cultivating growth-arrested cells without PHE- and TYR-supplementation drastically increased volumetric activity, achieving similar IND titers in much shorter timeframes.

*C. glutamicum* has also been engineered to synthesize various TRP-derivatives (e.g., halogenated derivatives) with diverse chemical and pharmacological applications. However, since a comprehensive review on this topic has been published recently, these compounds will not be discussed further here (Xiao et al. 2023).

Another compound with many applications in the cosmetic and food industries is 2-phenylethanol (2PE), an aromatic alcohol with a rose-like smell, which can be synthesized from the PHE-precursor phenylpyruvate (PPA) (Fig. 2) (Zhu et al. 2023). To achieve this, a heterologous Ehrlich pathway, facilitating the decarboxylation and reduction of PPA, was functionally integrated into an evolved PHE-producing *C. glutamicum* strain. For the Ehrlich pathway two genes—*aro10*, encoding 2-ketoisovalerate decarboxylase (KID) from *S. cerevisiae*, and *yahK* encoding alcohol dehydrogenase (ADH) originating from *E. coli*—were heterologously expressed. With additional modifications, the best variant produced up to 3.23 g L^−1^ 2PE from glucose in shake flask cultivations. When equipped with the isomerase pathway for xylose assimilation and a xylose transporter gene from *E. coli*, the *C. glutamicum* strain produced up to 3.55 g L^−1^ 2PE using xylose as sole carbon and energy source. The same strain accumulated 3.28 g L^−1^ 2PE from stalk hydrolysate as second-generation feedstock.

The Ehrlich pathway can also be utilized in a *C. glutamicum* variant engineered for TYR-production to synthesize tyrosol (TYO) and its glycosylated derivative, salidroside (SAL) (Fig. 2). SAL was first identified in raspberry extracts during a screening for bioactive compounds with potential therapeutic effects against Huntington’s disease (Kallscheuer et al. 2019b). To enable microbial SAL production from TYR, the Ehrlich pathway was reconstructed in *C. glutamicum*. Before decarboxylation and reduction to TYO, deamination of TYR to hydroxyphenylpyruvate (HPPY) was required, a step which was potentially catalyzed by the endogenous transaminases (ATs) AroT and/or IlvE (Marienhagen et al. 2005). However, even with TYR supplementation, only 0.15 mg L^−1^ SAL could be produced in shake flask cultivations. A more successful approach involved the direct supplementation of TYO, followed by its glycosylation using a UDP-dependent glycosyltransferase (UDPG) from *Oryza sativa*. Under these biotransformative production conditions, SAL production reached 9.7 g L^−1^. More recently, Junker and coworkers also followed the idea to introduce the Ehrlich pathway into *C. glutamicum* to produce TYO from TYR (Junker et al. 2025). The best strain variant allowed for a product titer of 1.3 g L^−1^ TYO, also relying on endogenous transaminases to catalyze the conversion of TYR to HPPY prior to decarboxylation and reduction to TYO.

β-arbutin (ARB), can also be found in plants such as pears, cranberries, and bearberry. ARB is a hydroquinone glucoside with antioxidant, anti-inflammatory, antimicrobial, and anticancer properties (Saeedi et al. 2021). The initial step of ARB synthesis from CHO was established in *C. glutamicum* by introducing the previously mentioned CHPL UbiC, which facilitates the conversion of CHO to 4HB (Fig. 1) (Zhang et al. 2024). Heterologous expression of a gene encoding 4-hydroxybenzoate-1-hydroxylase (4HB1H), identified in *Candida parapsilosis* CBS604 enabled the reductive decarboxylation of 4HB to hydroquinone (HQ). HQ was then glycosylated by an UDP-dependent hydroquinone glycosyltransferase (HQG) derived from *Rauvolfia serpentin*, ultimately yielding ARB. Since these genes were introduced into a *C. glutamicum* variant lacking CM- or ANS-activities due to deletions of *csm* and *trpE*, a complex medium was required to enable the production of 7.94 g L^−1^ ARB in shake flasks.

However, TYR is not only an intermediate of TYO- and SAL-biosynthesis, but also a direct precursor of various other aromatic compounds of biotechnological interest. Tyramine (TYRA), the decarboxylation product of TYR has garnered attention as starting material for the production of high-performance thermoplastics, pharmaceutically relevant compounds and hydrogels (Fig. 2) (Schulz et al. 2019; Chen et al. 2023). Heterologous expression of a tyrosine decarboxylase (TYD) gene from *Levilactobacillus brevis* enabled the production of up to 1.6 g L^−1^ TYRA from glucose in a *C. glutamicum* strain engineered for TYR production during shake flask cultivations (Poethe et al. 2024). Under the same conditions, introduction of the isomerase pathway into the best strain also facilitated TYRA production from xylose (1.2 g L^−1^). Batch fermentations with glucose or xylose in bioreactors allowed for faster growth and product formation but resulted in lower final TYRA titers (1 g L^−1^ TYRA for both carbon sources). The decarboxylation of TYR to TYRA also offers the possibility to produce TYO from TYRA via oxidative deamination in a single step (Junker et al. 2025) (Fig. 2). To achieve this, a gene for a tyramine oxidase (TO) from *Kocuria rhizophila* was introduced into a strain expressing a gene encoding a TYD. This strain accumulated up to 1.87 g L^−1^ TYRA. In co-cultivations of two *C. glutamicum* variants either expressing a gene for a TO or a TYD, the TYO titer was increased to 1.95 g L^−1^ TYO.

The hydroxylation of the aromatic ring of TYR by tyrosinase (TYRO) in the presence of oxygen yields 3,4-dihydroxyphenyl-l-alanine (DOPA), the most commonly used treatment of Parkinson’s disease (Fig. 2) (Chávez-Béjar et al. 2012). Heterologous expression of a TYRO-encoding gene from *Ralstonia solanacearum* in wild-type *C. glutamicum* cells enabled the production of 0.26 g L^−1^ DOPA from 1 g L^−1^ TYR when using glucose and xylose as carbon sources (Kurpejović et al. 2021). In these experiments, optimal concentrations of 0.4 mM copper sulfate—essential for TYRO activity as a copper-containing enzyme—and 0.2 mM thymol to prevent undesired DOPA oxidation were determined for cultivations. Additionally, whole-cells pre-grown on glucose and producing TYRO were used for biotransformations of TYR to DOPA in distilled water without any buffering agent. Under these conditions, up to 0.4 g L^−1^ DOPA was produced in the presence of 0.4 mM ascorbic acid to prevent any undesired DOPA oxidation.

The non-oxidative deamination of TYR by tyrosine ammonia-lyases (TALs) yields *para*-coumarate (CA, or 4-hydroxycinnamate), a phenylpropanoid believed to possess several bioactive properties, including antioxidant, antimicrobial, or anti-inflammatory effects (Fig. 2) (Zhu et al. 2024). Heterologous expression of a TAL-encoding gene from *Flavobacterium johnsoniae* enabled the conversion of endogenously provided TYR to CA in the previously discussed *C. glutamicum* DelAro^5^ variant unable to catabolize aromatics (Mutz et al. 2023). The accumulation of ANT as a major byproduct was eliminated by reducing ANS activity through targeted mutagenesis, thereby circumventing TRP auxotrophy. Subsequently, PHE biosynthesis was reduced by replacing the ATG start codon of *pheA*, the PDH-encoding gene by the less preferred GTG, and PEP availability was improved to further increase CA accumulation (Fig. 2). As a result, a maximum titer of 0.7 g L^−1^ CA was achieved in defined medium.

## (Poly)phenols and aromatic polyketides

**Fig. 3:**
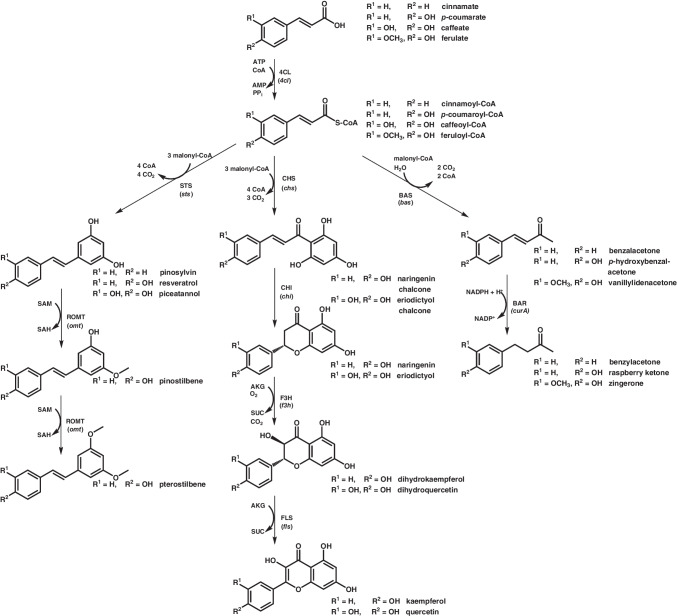
Schematic overview of the heterologous pathways leading to different plant polyketides, which have been functionally implemented into *C. glutamicum*. Precursors, co-substrates, intermediates and products (For the sake of consistency, all carboxyl groups are depicted in their protonated state): AKG, α-ketoglutarate; SUC, succinate; SAH, *S*-adenosylhomocysteine; SAM, *S*-adenosylmethionine. Enzymes (genes): 4CL (*4cl*), 4-coumarate: CoA ligase (e.g., *Petroselinum crispum*); BAR (*curA*), benzalacetone reductase (*E. coli*); BAS (*bas*), benzalacetone synthase (*Rheum palmatum*); CHI (*chi*), chalcone isomerase (*Petunia* × *hybrida*); CHS (*chs*), chalcone synthase (*Petunia* × *hybrida*); F3H (*f3h*), flavanone-3-hydroxylase (*Petunia* × *hybrida*); FLS (*fls*), flavonol synthase (*Populus deltoides*); ROMT (*omt*), resveratrol-di-*O*-methyltransferase (*Vitis vinifera*); STS (*sts*), stilbene synthase (*Arachis hypogaea*)

In plants, CA and other phenylpropanoids such as cinnamate, caffeate, or ferulate serve as precursors for more complex (poly)phenols such as flavonoids and stilbenoids (Fig. 3) (Marienhagen and Bott 2013). These compounds can also be classified as polyketides since their synthesis involves polyketide synthases (PKSs). Several thousand different (poly)phenols have been identified and can be categorized into chemically distinct groups, including stilbenoids, flavonoids, and phenylbutanoids—many of which have potential applications in preventing or treating cancer, cardiovascular diseases, and neurodegenerative disorders (Milke et al. 2018). However, plants usually contain complex mixtures of chemically similar polyphenolic compounds, making it challenging to isolate individual compounds in large quantities. In contrast, integrating biosynthetic pathways for plant polyphenols into microorganisms enables the production of individual polyphenols as chemically distinct compounds. This approach allows for large-scale synthesis and easier isolation. In recent years, *C. glutamicum* has been engineered to produce various biotechnologically and pharmaceutically relevant plant (poly)phenols.

The first significant step in engineering *C. glutamicum* for polyphenol production was achieved in 2016 with the construction of a platform strain featuring the deletion of four gene clusters comprising 21 genes involved in the catabolism of aromatic compounds (Kallscheuer et al. 2016b). Among these were four genes of the *phd* gene cluster discovered shortly before, which plays a crucial role in the degradation of phenylpropanoids via a CoA-dependent β-oxidative deacetylation pathway (Kallscheuer et al. 2016a). To facilitate polyphenol biosynthesis, codon-optimized, plant-derived genes were heterologously expressed in this *C. glutamicum* variant. These included a 4-coumarate:CoA ligase (4CL) from parsley (*Petroselinum crispum*), responsible for the CoA activation of supplemented phenylpropanoids, and a stilbene synthase (STS) from peanut (*Arachis hypogaea*), a type III PKS. STSs catalyze the sequential condensation of three malonyl-CoA units with the CoA-activated phenylpropanoid, followed by cyclization (van Summeren-Wesenhagen and Marienhagen 2013). This engineered pathway enabled the production of pinosylvin, resveratrol (RES), and piceatannol from supplemented cinnamate, CA, and caffeate, respectively (Fig. 3) (Kallscheuer et al. 2016b). Unlike most other bacteria, *C. glutamicum* possesses a type I fatty acid synthase, which is a eukarotic-type multienzyme, into which all activities required for fatty acid elongation are integrated (Schweizer and Hofmann 2004). The addition of 25 µM cerulenin, a fatty acid synthase inhibitor that selectively binds to the β-keto-acyl-ACP synthase subunit, thus blocking the interaction with malonyl-CoA, increases intracellular malonyl-CoA availability by shutting down de novo fatty acid biosynthesis. This enhanced stilbenoid production, yielding up to 0.16 g L^−1^ RES. Functional implementation of genes encoding chalcone synthase (CHS, *Petunia* × *hybrida*) and chalcone isomerase (CHI, *Petunia* × *hybrida*) in the same strain background enabled the biosynthesis of up to 0.04 g L^−1^ of the (2*S*)-flavanones naringenin (NAR) and eriodictyol (ERI) from supplemented CA and caffeate, respectively. Other attempts to produce ERI from TYR in *C. glutamicum* involved the supplementation of malonate as source for malonyl-CoA (Wu et al. 2022). To facilitate this, *matC* and *matB* from *Rhizobium trifolii*, encoding a malonate transporter and a malonyl-CoA synthetase, respectively, were introduced into *C. glutamicum*. The resulting strain, also expressing genes for a 4CL from *P. crispum*, a CHS from *Petunia* × *hybrida* and a CHI from *Medicago sativa* accumulated 0.02 g L^−1^ NAR in complex medium containing 20 g L^−1^ glucose, 2 g L^−1^ malonate, and 0.5 g L^−1^ TYR. Subsequently, the *hpaBC* genes from *E. coli* encoding 4-hydroxyphenylacetate-3-hydroxylase were expressed in *C. glutamicum* to convert NAR to ERI. Under the same cultivation conditions, up to 0.01 g L^−1^ ERI was produced. Kallscheuer and colleagues made also initial steps towards polyphenol production from glucose by integrating DAHPS from *E. coli* to enhance carbon flux into the shikimate pathway (Kallscheuer et al. 2016b). Along with the already mentioned TAL from *F. johnsoniae* to facilitate the conversion of endogenous TYR to CA up to 0.06 g L^−1^ RES could be obtained with the resulting strain in shake flask cultivations and in the presence of 25 µM cerulenin. Subsequently, the polyphenol portfolio accessible via *C. glutamicum* was expanded by decorating stilbenoid and flavonoid core structures by *O*-methylation or hydroxylation providing access to more stable compounds of higher commercial interest (Kallscheuer et al. 2017b). Heterologous expression of a gene for an *O*-methyltransferase (ROMT) from *Vitis vinifera* in a RES-producing *C. glutamicum* strain allowed for the synthesis of trace amounts of mono-*O*-methylated pinostilbene and 0.04 g L^−1^ di-*O*-methylated pterostilbene from supplemented CA (Fig. 3). Furthermore, the expression of heterologous genes encoding 2-oxoglutarate-dependent dioxygenases in (2*S*)-flavanone-producing *C. glutamicum* strains enabled the production of flavanonols and flavonols starting from the phenylpropanoids CA and caffeate (Fig. 3) (Kallscheuer et al. 2017b). The targeted flavonols kaempferol and quercetin were produced with maximum titers of 0.02 g L^−1^ and 0.01 g L^−1^, respectively. Notably, efforts were made to produce polyphenols independently from the shikimate pathway (Kallscheuer et al. 2017c). To achieve this, a synthetic pathway utilizing inexpensive supplemented benzoic acids, such as 4HB, was introduced into *C. glutamicum*. This metabolic strategy mimics a reversed β-oxidative phenylpropanoid degradation pathway, eliminating the need for ammonia lyase activity (Kallscheuer et al. 2017a). Using this approach, RES titers of 0.005 g L^−1^ RES were achieved from 5 mM 4HB.

Hitherto, most of these experiments were conducted with the costly supplementation of cerulenin to increase the malonyl-CoA pool, as availability of this two-carbon donor molecule represents the key bottleneck in microbial polyphenol synthesis (Milke and Marienhagen 2020). Consequently, subsequent metabolic engineering efforts focused on enhancing malonyl-CoA availability independent of exogenous fatty acid inhibitors. Initial experiments demonstrated that the sole overexpression of endogenous *accBCD1* genes encoding the native acetyl‐CoA carboxylase was insufficient to enhance polyphenol production in *C. glutamicum* (Milke et al. 2019a). As an alternative, intracellular acetyl‐CoA availability was increased by reducing the flux into the TCA cycle through reduction of citrate synthase activity by promoter replacement of *gltA* coding for this enzyme. In defined medium, these strains accumulated 0.02 g L^−1^ NAR or 0.11 g L^−1^ RES from glucose without supplementation of phenylpropanoid precursors or cerulenin. In this study, the *fasR* gene was deleted. *FasR* encodes a transcriptional repressor, which negatively regulates the expression of *accBCD1* and of the genes for two fatty acid synthases (*fasIA* and *fasIB*). However, the inactivation of *fasR* led to a severe growth defect, likely due to deregulated fatty acid synthesis consuming cellular resources (Milke et al. 2019a). To address this issue, only the FasR-binding operator sequences upstream of the *accBCD1* open reading frames were mutated, while maintaining the natural FasR-mediated regulation of *fasIA* and *fasIB* (Milke et al. 2019b). In combination with other modifications, the resulting strain exhibited a significantly increased intracellular malonyl-CoA pool, as confirmed by intracellular LC–MS/MS measurements and enhanced product formation. Notably, the resulting *C. glutamicum* variant named M-CoA, equipped with a plasmid enabling RES production from supplemented CA, could be successfully applied in co-cultivation with the aforementioned CA-producing *C. glutamicum* variant, yielding 0.03 g L^−1^ RES from glucose (Mutz et al. 2024).

Subsequently, *C. glutamicum* M-CoA was successfully employed for the microbial production of flavoring phenylbutanoids (Milke et al. 2020). One such compound, 4-(4-hydroxyphenyl)butan-2-one—commonly known as raspberry ketone—is responsible for the typical scent and flavor of raspberries (Guo et al. 2021). The chemical synthesis of nature-identical raspberry ketone is well established, as this compound is widely used in the flavoring of food, beverages, and perfumes. However, the combination of high demand for natural raspberry ketone and its low natural abundance in raspberries makes it expensive natural flavoring components. *C. glutamicum* was engineered for raspberry ketone synthesis from supplemented CA by combining the already mentioned 4CL from *P. crispum* with the benzalacetone synthase (BAS) from Chinese rhubarb (*Rheum palmatum*) yielding *p*-hydroxybenzalacetone (Fig. 3) (Milke et al. 2020). For its subsequent reduction to raspberry ketone, the NADPH-dependent curcumin/dihydrocurcumin reductase CurA from *E. coli* was employed as it provides a hitherto unknown benzalacetone reductase (BAR) activity. The engineered strain accumulated up to 0.1 g L^−1^ raspberry ketone. Additionally, supplementing ferulate or cinnamate instead of CA enabled the biosynthesis of two other naturally occurring flavor compounds, zingerone (0.07 g L^−1^) and benzylacetone (0.01 g L^−1^), via the same pathway (Fig. 3).

**Fig. 4:**
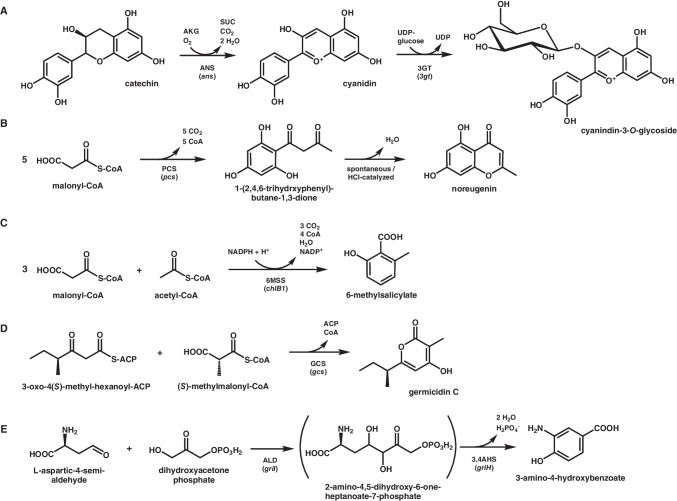
Schematic overview of the enzymatic steps leading to other aromatic compounds accessible by *C. glutamicum*: **A** cyaniding-3-*O*-glycoside, **B** the type I polyketide noreugenin, the type III polyketides **C** 6-methylsalicylate and **D** germicidin C, and **E** the non-shikimate pathway-derived aromatic compound 3-amino-4-hydroxybenzoate. 2-amino-4,5-dihydroxy-6-one-heptanoate-7-phosphate is the probable product of the aldolase GriI, but it has not been independently characterized. Precursors, co-substrates, intermediates and products (for the sake of consistency, all carboxyl groups are depicted in their protonated state): ACP, acyl carrier protein; AKG, α-ketoglutarate; SUC, succinate; UDP, uracil-diphosphate; UDP-glucose, uracil-diphosphate glucose. Enzymes (genes): 3,4AHS (*griH*), 3-amino-4-hydroxybenzoate synthase (*Streptomyces griseus*); 3GT (*3gt*), flavonoid 3-*O*-glucosyltransferase (*Arabidopsis thaliana*); 6MSS (chlB1), 6-methylsalicylic acid synthase (*Streptomyces antibioticus*); ALD (*griH*), aldolase (*Streptomyces griseus*); ANS (*ans*), anthocyanidin synthase (*Petunia hybrida*); GCS (*gcs*), germicidin synthase (*Streptomyces coelicolor*); PCS (*pcs*), pentaketide chromone synthase (*Aloe arborescens*)

In 2018, Zha et al. demonstrated that *C. glutamicum* can convert the supplemented flavonoid catechin to the anthocyanin cyanidin 3-*O*-glucoside (Zha et al. 2018). To achieve this, genes encoding anthocyanidin synthase (ANS) from *Petunia* × *hybrida* and flavonoid 3-*O*-glucosyltransferase (3GT) from *Arabidopsis thaliana* were co-expressed in *C. glutamicum*. ANS, a 2-oxoglutarate- and iron-dependent oxygenase, catalyzes catechin oxidation to cyanidin, while the 3GT facilitates cyanidin glycosylation, yielding the more stable cyanidin 3-*O*-glucoside (Fig. 4A). Further process optimization and enhanced UDP-glucose availability enabled the production of 0.04 g L^−1^ cyanidin 3-*O*-glucoside from 0.5 g L^−1^ catechin in complex medium.

During several fed-batch fermentations in bioreactors for plant polyphenol production, it became evident that maintaining product stability at elevated oxygen concentrations poses a significant challenge (Braga et al. 2018). In this context, ISPR, particularly through a biphasic extractive strategy, offers a promising approach to protect polyphenol products from oxidative degradation (Tharmasothirajan et al. 2021). Additionally, ISPR serves as an effective method to mitigate cytotoxic effects associated with high product concentrations while enhancing overall process performance. A solvent screening identified tributyrin as the most biocompatible solvent for *C. glutamicum*, making it the preferred choice for biphasic extraction due to its favorable partitioning and solubility properties for RES (Tharmasothirajan et al. 2021). In bioreactor studies, biphasic cultivation with tributyrin enabled a RES titer of 1.71 g L^−1^, with approximately 64% of the stilbenoid successfully recovered from the tributyrin phase at an elevated pH of 12.

## Other aromatic compounds of biotechnological interest

All aromatic products described thus far are derived from intermediates or CHO as end product of the shikimate pathway, or the biosynthetic routes leading to the three aromatic amino acids. However, PKSs are not only relevant for polyphenol synthesis from phenylpropanoids as previously discussed, but can also be employed to directly synthesize aromatic compounds from acetyl-CoA, malonyl-CoA and other activated aliphatic precursors.

The first example is the chromone noreugenin, a pharmacologically relevant plant pentaketide synthesized from malonyl-CoA by medicinal plant plants such as *Aloe arborescens* (Abe et al. 2005). To establish noreugenin biosynthesis in *C. glutamicum*, the above-mentioned malonyl-CoA providing variant *C. glutamicum* M-CoA was used as a chassis (Milke et al. 2019b). For product synthesis, only a single gene from *A. aborescens*, encoding pentaketide chromone synthase (PCS), needed to be heterologously expressed. This type III PKS catalyzes the iterative decarboxylation and condensation of five malonyl-CoA molecules yielding noreugenin (Fig. 4B). Initially, only the accumulation of the C1/C6 cyclized intermediate 1-(2,4,6-trihydroxyphenyl)-butane-1,3-dione was detected. However, acidification of the culture broth post-cultivation enabled full cyclization, resulting in the accumulation of 0.05 g L^−1^ of the bicyclic end product, noreugenin.

*C. glutamicum* M-CoA was also the strain of choice for establishing 6-methylsalicylic acid (6MSA) synthesis (Kallscheuer et al. 2019a). This chemically simple aromatic compound is a building block in the biosynthesis of several antibiotics, including pactamycin and polyketomycin (Daum et al. 2009; Ito et al. 2009). Establishing microbial 6MSA synthesis in *C. glutamicum* marked the first instance of introducing a large multi-domain type I PKS in this host. Specifically, the 6MSA synthase (6MSS) ChlB1 from *Streptomyces antibioticus*—a 5.3 kbp-gene encoding a 186 kDa protein—catalyzes the conversion of acetyl-CoA and three molecules of malonyl-CoA into 6MSA in consecutive steps (Fig. 4C). Initial challenges related to protein folding were overcome by translation fusion of ChlB1 to the C-terminus of the maltose-binding protein MalE from *E. coli*, which supported proper expression. Interestingly, the 6MSS exhibited activity even in the absence of a heterologous dedicated 4′-phosphopantetheinyl transferase (PPTase), typically required for post-translational activation of type I PKSs. This unexpected finding led to the discovery that the endogenous PPTase PptA of *C. glutamicum* can also activate ChlB1Sa. The best-performing strain accumulated up to 0.04 g L^−1^ 6MSA within 48 h of cultivation. Further experiments revealed that PptA can also activate non-ribosomal peptide synthetases, positioning *C. glutamicum* as a promising microbial platform for the production of other fine chemicals and medicinal drugs (Kallscheuer et al. 2019a).

Germicidins, a class of 2-pyrones from the sponge-associated *Streptomyces* sp., exhibit both, aromatic and aliphatic characteristics, and possess significant, specific inhibitory activity against human hexokinase, an abundant enzyme in cancer cells (Bai et al. 2021). Zhan and colleagues established germicidin C-synthesis in *C. glutamicum* by introducing germicidin synthase (GCS), a type III PKS from *Streptomyces coelicolor* (Zhan et al. 2023)*.* GCS catalyzes the condensation of 3-oxo-4(*S*)-methyl-hexanoyl-ACP and methylmalonyl-CoA to produce germicidin (Fig. 4D). However, the accumulation of propionyl-CoA and methylmalonyl-CoA leads to growth inhibition, but introduction of GCS as methylmalonyl-CoA-dependent PKS can relieve this inhibitory effect. To further enhance germicidin production, ALE was employed, leveraging the fitness advantage conferred by polyketide biosynthesis in the presence of propionate. This approach led to an improved germicidin titer of 0.01 g L^−1^.

In addition to PKSs, other enzymes also enable access to aromatic compounds independently of the shikimate pathway. The only example in *C. glutamicum* thus far is the production of the bioplastic precursor 3-amino-4-hydroxybenzoate (3,4-AHBA). This compound, an intermediate of grixazone biosynthesis in *Streptomyces griseus*, is synthesized directly from l-aspartate-4-semialdehyde and dihydroxyacetone phosphate via the activities of two enzymes: GriI and GriH (Fig. 4E) (Suzuki et al. 2006). GriI catalyzes an aldol condensation between l-aspartate-4-semialdehyde and dihydroxyacetone phosphate, and GriH converts the resulting C7 metabolite into 3,4-AHBA. A *C. glutamicum* l-lysine producing strain, heterologously expressing *S. griseus griI* and *griH* successfully produced 1 g L^−1^ 3,4-AHBA from sweet sorghum juice (Kawaguchi et al. 2015). Interestingly, production titers from sweet sorghum juice rich in amino acids were fivefold higher than from pure sucrose. Supplementation experiments suggested that l-leucine specifically enhanced 3,4-AHBA production, likely by increasing pyruvate availability for l-aspartate-4-semialdehyde biosynthesis. A subsequent study showed that 3,4-AHBA production improved under low dissolved oxygen conditions (Kawaguchi et al. 2021). Comparative metabolic profiling at different oxygen levels revealed accumulation of different organic acids including TCA-cycle intermediates and lactate. An engineered strain lacking lactate dehydrogenase activity accumulated up to 5.6 g L^−1^ 3,4-AHBA in glucose fed-batch cultures, likely due to enhanced pyruvate availability.

## Conclusions and challenges

The portfolio of aromatic chemicals, which can be produced from renewable resources through microbial fermentation has significantly expanded in recent years, and *C. glutamicum*, already well known in the industry as an amino acid producer, has been developed to a versatile platform organism for the production of these valuable compounds.

In particular, the presented *C. glutamicum* chassis strains providing aromatic precursor molecules derived from the shikimate pathway or CoA-activated thioesters such as malonyl-CoA represent excellent hosts for the functional implementation of biosynthetic gene clusters from various different sources. Very recently, *C. glutamicum* M-CoA, mentioned several times in this review, was used to express genes encoding six selected type III PKSs of unknown function from different planctomycetes to screen for novel activities (Milke et al. 2024). In these experiments, several enzymes involved in the synthesis of long-chain alkylresorcinols—compounds with potential functions as antibiotics or electron carriers—were identified.

Further improvements of *C. glutamicum*–based cell factories for aromatics are necessary to enhance the cost-competitiveness of biotechnologically produced aromatic chemicals compared to conventional petroleum-derived compounds. A key focus should be on suitable metabolic engineering strategies. Many examples presented in this review rely heavily on the genetically induced auxotrophies for one or more aromatic amino acids, to reroute the carbon flux and maximize product titers (Table 1). However, depending on the targeted product, such strain designs would be economically unfeasible for large-scale industrial bioprocesses, as they would require costly supplementation of essential compounds. Similarly, the choice of the cultivation medium plays a crucial role—it should be as simple as possible and should fit to subsequent downstream processing strategies. The use of complex medium components such as peptone, tryptone or yeast extract, which were included in some studies reviewed here, is unlikely to be viable in scaled-up bioprocesses (Table 1). Notably, avoiding auxotrophies and utilizing defined media without complex additives would also improve the comparability (and likely also the reproducibility) of scientific studies on the production of small aromatic compounds with *C. glutamicum*.

**Table 1:** Overview of aromatic compounds accessible with *Corynebacterium glutamicum*

**Aromatic compound**	**Cultivation medium**^**a**^	**Type of cultivation**	**Titer** [g L^−1^]	**Cultivation time** [h]	**Reference**
**Shikimate pathway intermediates and aromatic amino acids**					
Shikimate	Complex medium; suc, 38	Batch, shake flask	7.4	96	Zhang et al. 2015
Shikimate	Complex medium; suc	Fed-batch, bioreactor	11.3	70	Zhang et al. 2015
Shikimate	Defined medium; glu	Fed-batch, bioreactor, growth-arrested cells	141	48	Kogure et al. 2016
Shikimate	Defined medium; glu, xyl, ara	Fed-batch, bioreactor, growth-arrested cells	137	48	Kogure et al. 2016
Shikimate	Complex medium; Glu, 50; l-phe, 0.1; l-trp; 0.1; l-tyr 0.1	Batch, unspecified vessel	13.1	72	Sato et al. 2020
Shikimate	Complex medium; cel, 50; l-phe, 0.1; l-trp; 0.1; l-tyr 0.1	Batch, unspecified vessel	13.8	72	Sato et al. 2020
Anthranilate	Defined CGXII-medium; glu; l-trp supplementation	Fed-batch, bioreactor	26.4	84	Luo et al. 2019
Anthranilate	Defined CGXII-medium; glu; xyl	Fed-batch, bioreactor	5.9	192	Mutz et al. 2024
l-Phenylalanine	Complex medium; glu	Fed-batch, bioreactor	15.8	80	Zhang et al. 2015
l-Phenylalanine	Complex medium; glu, 80	Batch, shake flask	6.11	72	Tachikawa et al. 2024
l-Tryptophan	Defined CGXII medium; glu, 40; l-phe, 0.25; l-tyr, 0.25	Batch, shake flask	2.14	48	Mindt et al. 2023
l-Tyrosine	Defined CGXII medium; glu, 40; l-tyr, 0.5 mM	Batch, shake flask	3.2	48	Kurpejović et al. 2023
l-Tyrosine	Defined CGXII medium; glu, 10; xyl, 30; l-tyr, 0.5 mM	Batch, shake flask	3.6	48	Kurpejović et al. 2023
**Shikimate pathway-derived compounds**					
Protocatechuate	Complex medium; glu	Fed-batch, bioreactor	1.14	120	Okai et al. 2016
Protocatechuate	Defined CGXII medium; glu, 40	Batch, shake flask	2	72	Kallscheuer and Marienhagen 2018
Protocatechuate	Defined BT medium, glu	Fed-batch, bioreactor, growth-arrested cells	82.7	32	Kogure et al. 2021
Protocatechuate	Defined CGXII medium, xyl, 40	Batch, shake flask	9.6	100	Labib et al. 2021
Protocatechuate	Defined CGXII medium, glu	Fed-batch, bioreactor	16.5	130	Labib et al. 2021
2-hydroxybenzoate (salicylic acid)	Defined CGXII medium; glu, 40	Batch, shake flask	0.01	72	Kallscheuer and Marienhagen 2018
3-hydroxybenzoate	Defined CGXII medium; glu, 40	Batch, shake flask	0.3	72	Kallscheuer and Marienhagen 2018
4-hydroxybenzoate	Defined CGXII medium; glu, 40	Batch, shake flask	3.3	72	Kallscheuer and Marienhagen 2018
4-hydroxybenzoate	Complex medium; glu; l-phe, 0.9; l-trp, 0.7; l-tyr, 0.8	Fed-batch, bioreactor	19	65	Purwanto et al., 2018
4-hydroxybenzoate	Defined medium; glu	Fed-batch, bioreactor, growth-arrested cells	36,6	24	Kitade et al. 2018
4-hydroxybenzaldehyde	Complex medium; glu, 80	Batch, shake flask	1.36	48	Kim et al. 2022
Protocatechuate aldehyde	Complex medium; glu, 80	Batch, shake flask	1.18	48	Kim et al. 2022
Vanillin	Complex medium; glu, 80; l-met, 0.5	Batch, shake flask	0.31	48	Kim et al. 2022
Vanillin	Complex medium; glu, 85.7; vanillic acid, 42.9	Biotransformation of vanillic acid, jar reactor	21	45	Matsuzawa et al. 2024
4-aminobenzoate	Complex medium; glu	Fed-batch, bioreactor	43.1	48	Kubota et al. 2016
4-amino-3-hydroxybenzoate	Complex medium; glu	Fed-batch, bioreactor	13.5	72	Nonaka et al. 2023
Methylanthranilate	Defined CGXII-medium; glu; l-trp supplementation	Fed-batch, bioreactor	5.74	84	Luo et al. 2019
Indole	Defined CGXII medium; glu, 40; l-phe, 0.25; l-trp, 0.25;	Batch, shake flask	0.67	48	Ferrer et al. 2022
	l-tyr, 0.25; in situ product removal with tributyrin, 20% (vol/vol)				
Indole	Defined CGXII medium; glu, 10; l-trp, 10	Biotransformation of l-trp, bioreactor	5.7	24	Mindt et al. 2022
	In situ product removal with dibutyl sebacate, 20% (vol/vol)				
Indole	Defined CGXII medium; glu, 40; l-phe, 0.25; l-tyr, 0.25;	Batch, shake flask	1.38	70	Mindt et al. 2023
	In situ product removal with tributyrin, 20% (vol/vol)				
2-phenylethanol	Complex medium; glu, 60	Batch, shake flask	3.23	50	Zhu et al. 2023
2-phenylethanol	Complex medium; xyl, 60	Batch, shake flask	3.55	60	Zhu et al. 2023
2-phenylethanol	Complex medium; corn stalk hydrolysate (glu, 41; xyl, 19)	Batch, shake flask	3.28	48	Zhu et al. 2023
Tyrosol	Defined medium; glu; l-phe, 0.5 mM	Batch, shake flask	1.3	160	Junker et al. 2025
Tyrosol	Defined medium; glu; l-phe, 0.5 mM	Batch, shake flask	1.87	160	Junker et al. 2025
Tyrosol	Defined medium; glu; l-phe, 0.5 mM	Batch, shake flask	1.95	160	Junker et al. 2025
Salidroside	Defined CGXII medium; l-tyr, 20 mM	Batch, shake flask	0.15	72	Kallscheuer et al. 2019b
Salidroside	Defined CGXII medium; tyrosol, 40 mM	Biotransformation of tyrosol, shake flask	9.7	100	Kallscheuer et al. 2019b
β-arbutin	Complex medium, glu 80, insufficient information on media supplements	Batch, shake flask	7.94	72	Zhang et al. 2024
Tyramine	Defined CGXII medium; glu, 40; l-phe, 0.5 mM	Batch, shake flask	1.6	72	Poethe et al. 2024
Tyramine	Defined CGXII medium; xyl, 40; l-phe, 0.5 mM	Batch, shake flask	1.2	72	Poethe et al. 2024
Tyramine	Defined CGXII medium; glu, 40; l-phe, 1 mM; l-trp, 1 mM	Batch, bioreactor	1	36	Poethe et al. 2024
Tyramine	Defined CGXII medium; xyl, 40; l-phe, 1 mM; l-trp, 1 mM	Batch, bioreactor	1	72	Poethe et al. 2024
3,4-dihydroxyphenyl-l-alanine (l-DOPA)	Defined CGXII medium; glu, 10; xyl, 3; l-tyr, 1	Batch, shake flask	0.36	72	Kurpejović et al. 2021
3,4-dihydroxyphenyl-l-alanine (l-DOPA)	Water; l-tyr, 1	Biotransformation, 15-ml test tubes	0.4	48	Kurpejović et al. 2021
*p*-coumarate	Defined CGXII medium; glu, 40	Batch, shake flask	0.66	72	Mutz et al. 2024
**Aromatic polyketides**					
Resveratrol	Defined CGXII medium; glu, 40; *p-*ca, 5 mM; cer, 0.025 mM	Biotransformation of *p*-coumarate, shake flask	0.16	72	Kallscheuer et al. 2016b
Resveratrol	Defined CGXII medium; glu, 40; cer, 0.025 mM	Batch, shake flask	0.06	72	Kallscheuer et al. 2016b
Resveratrol	Defined CGXII medium; glu, 40	Batch, shake flask	0.11	72	Milke et al. 2019a
Resveratrol	Defined CGXII medium; glu; *p*-ca, 5 mM	Fed-batch, bioreactor	1.71	72	Tharmasothirajan et al. 2021
	In situ product removal with tributyrin, 10% (vol/vol)				
Resveratrol	Defined CGXII medium; glu; 40; co-cultivation of	Batch, shake flask	0.03	72	Mutz et al. 2024
	*p*-ca and resveratrol prod. *C. glutamicum* variants				
Pinostilbene	Defined CGXII medium; glu, 40; *p-*ca, 5 mM; cer, 0.025 mM	Biotransformation of *p*-coumarate, shake flask	0.01	144	Kallscheuer et al. 2017b
Pterostilbene	Defined CGXII medium; glu, 40; *p*-ca, 5 mM; cer, 0.025 mM	Biotransformation of *p*-coumarate, shake flask	0.04	144	Kallscheuer et al. 2017b
Pinosylvin	Defined CGXII medium; glu, 40; cinnamate, 5 mM; cer, 0.025 mM	Biotransformation of cinnamate, shake flask	0.12	72	Kallscheuer et al. 2016b
Piceatannol	Defined CGXII medium; glu, 40; caffeate, 5 mM; cer, 0.025 mM	Biotransformation of caffeate, shake flask	0.06	72	Kallscheuer et al. 2016b
Naringenin	Defined CGXII medium; glu, 40; *p*-ca, 5 mM; cer, 0.025 mM	Biotransformation of *p*-coumarate, shake flask	0.04	72	Kallscheuer et al. 2016b
Naringenin	Defined CGXII medium; glu, 40	Batch, shake flask	0.02	72	Milke et al. 2019a
Naringenin	Complex medium; glu, 20; l-tyr, 0.5; malonate, 2	Batch, shake flask	0.01	72	Wu et al. 2022
Dihydrokaempferol	Defined CGXII medium; glu, 40; *p*-ca, 5 mM; cer, 0.025 mM	Biotransformation of *p*-coumarate, shake flask	0.02	144	Kallscheuer et al. 2017b
Kaempferol	Defined CGXII medium; glu, 40; *p*-ca, 5 mM; cer, 0.025 mM	Biotransformation of *p*-coumarate, shake flask	0.02	144	Kallscheuer et al. 2017b
Eriodictyol	Defined CGXII medium; glu, 40; caffeate, 5 mM;	Biotransformation of caffeate, shake flask	0.04	72	Kallscheuer et al. 2016b
Eriodictyol	Complex medium; glu, 20; l-tyr, 0.5; malonate, 2	Batch, shake flask	0.01	72	Wu et al. 2022
Dihydroquerecetin	Defined CGXII medium; glu, 40; caffeate, 5 mM; cer, 0.025 mM	Biotransformation of *p*-coumarate, shake flask	0.01	144	Kallscheuer et al. 2017b
Quercetin	Defined CGXII medium; glu, 40; caffeate, 5 mM; cer, 0.025 mM	Biotransformation of *p*-coumarate, shake flask	0.01	144	Kallscheuer et al. 2017b
Raspberry ketone	Defined CGXII medium; glu, 40; *p*-ca, 5 mM	Biotransformation of *p*-coumarate, shake flask	0.1	72	Milke et al. 2020
Zingerone	Defined CGXII medium; glu, 40; ferulate, 5 mM	Biotransformation of ferulate, shake flask	0.07	72	Milke et al. 2020
Benzylacetone	Defined CGXII medium; glu, 40; cinnamate, 5 mM	Biotransformation of caffeate, shake flask	0.01	72	Milke et al. 2020
Cyanidin 3-*O*-glucoside	Complex medium; glu, 20; catechine 0.5	Biotransformation of catechin, shake flask	0.04	48	Zha et al. 2018
**Other non-shikimate pathway-derived aromatic compounds or polyketides**					
Noreugenin	Complex medium; glu, 40	Batch, shake flask	0.05	72	Milke et al. 2019b
6-methylsalicylic acid	Defined CGXII medium; glu, 40	Batch, shake flask	0.04	48	Kallscheuer et al. 2019a
Germicidin C	Defined CGXII medium, propionate	Incomplete information	0.01	80	Zhan et al. 2023
3-amino-4-hydroxybenzoate	Complex medium; sweet sorghum juice (suc, 40; glu, fru)	Batch, shake flask	1	72	Kawaguchi et al. 2015
3-amino-4-hydroxybenzoate	Defined CGXII medium; glu	Fed-batch, bioreactor	5.6	122	Kawaguchi et al. 2021

^a^A medium is considered to be “complex” when complex medium components such as yeast extract, peptone, or tryptone are added, or fed-batch cultivations only the carbon and energy source is indicated, not the amount of substrate added. All concentrations are given in g L^−1^ if not stated otherwise. Carbon sources used for *C. glutamicum* cultivations: *ara*, arabinose; *cel*, cellubiose; *fru*, fructose; *glu*, glucose; *rib*, ribose; *suc*, sucrose; *xyl*, xylose; *p-ca*, *p*-coumarate; *cer*, cerulenin

However, the development of suitable production strains for aromatic compounds requires accelerated strain construction and the characterization of numerous variants. To achieve this, biofoundries—advanced facilities that integrate automation, molecular biology, and computational tools—are essential for streamlining these processes. In such a setting, high-throughput technologies for tasks ranging from cell factory construction to cultivation and analytics, combined with machine learning to optimize the design-build-test-learn cycle, would unlock the full potential of *C. glutamicum*. Initial steps in this direction have already been taken (Kang et al. 2022; Rosch et al. 2024).

Last but not least, unconventional solutions are needed to address challenges such as substrate uptake, product export, or product toxicity. For instance, at higher product concentrations, most aromatic compounds are cytotoxic, negatively affecting cell integrity and transport processes, even in the case of such a robust host as *C. glutamicum*. Recently, a screening of free fatty acid supplements identified palmitelaidic acid and linoleic acid as cost-effective and suitable additives to mitigate cytotoxic effects of stilbenoid- and flavonoid production in *C. glutamicum* (Tharmasothirajan et al. 2023). These free fatty acids are not metabolized, but remain in the cell envelope, counteracting membrane damage by aromatic product accumulation. This strategy ultimately enabled up to a threefold increase in polyphenol titers in bioreactor cultivations. Given its cost-effectiveness, this approach could be a promising option for large-scale production of other membrane-active aromatic compounds of industrial value using *C. glutamicum*.

## Data Availability

No datasets were generated or analysed during the current study.

## References

[CR1] Abe I, Utsumi Y, Oguro S, Morita H, Sano Y, Noguchi H (2005) A plant type III polyketide synthase that produces pentaketide chromone. J Am Chem Soc 127:1362–1363. 10.1021/ja0431206 15686354 10.1021/ja0431206

[CR2] Averesch NJH, Krömer JO (2018) Metabolic engineering of the shikimate pathway for production of aromatics and derived compounds-present and future strain construction strategies. Front Bioeng Biotechnol 6. 10.3389/fbioe.2018.00032 10.3389/fbioe.2018.00032 PMC5879953 29632862

[CR3] Bai Y, Yi P, Zhang Y, Hu J, Wang Y, Ju J, Pan H (2021) Structure-based molecular networking for the target discovery of novel germicidin derivatives from the sponge-associated *Streptomyces* sp. 18A01. J Antibiot 74:799–806. 10.1038/s41429-021-00447-w 10.1038/s41429-021-00447-w 34272496

[CR4] Bang HB, Choi IH, Jang JH, Jeong KJ (2021) Engineering of *Escherichia coli* for the economic production L-phenylalanine in large-scale bioreactor. Biotechnol Bioprocess Eng 26:468–475. 10.1007/S12257-020-0313-1

[CR5] Braga A, Oliveira J, Silva R, Ferreira P, Rocha I, Kallscheuer N, Marienhagen J, Faria N (2018) Impact of the cultivation strategy on resveratrol production from glucose in engineered *Corynebacterium glutamicum*. J Biotechnol 265:70–75. 10.1016/j.jbiotec.2017.11.006 29141192 10.1016/j.jbiotec.2017.11.006

[CR6] Chávez-Béjar MI, Báez-Viveros JL, Martínez A, Bolívar F, Gosset G (2012) Biotechnological production of L-tyrosine and derived compounds. Process Biochem 47:1017–1026. 10.1016/J.PROCBIO.2012.04.005

[CR7] Chen M, He X, Lv J, xiao H, Tan W, Wang Y, Hu J, Zeng K, Yang G (2023) A new bio-based thermosetting with amorphous state, sub-zero softening point and high curing efficiency. Polymer 264:125518. 10.1016/J.POLYMER.2022.125518

[CR8] Cho JS, Luo ZW, Moon CW, Prabowo CPS, Lee SY (2024) Metabolic engineering of *Corynebacterium glutamicum* for the production of pyrone and pyridine dicarboxylic acids. Proc Natl Acad Sci U S A 121:e2415213121. 10.1073/PNAS.2415213121 39475655 10.1073/pnas.2415213121 PMC11551391

[CR9] Daum M, Peintner I, Linnenbrink A, Frerich A, Weber M, Paululat T, Bechthold A (2009) Organisation of the biosynthetic gene cluster and tailoring enzymes in the biosynthesis of the tetracyclic quinone glycoside antibiotic polyketomycin. ChemBioChem 10:1073–1083. 10.1002/CBIC.200800823 19266534 10.1002/cbic.200800823

[CR10] del Olmo A, Calzada J, Nuñez M (2017) Benzoic acid and its derivatives as naturally occurring compounds in foods and as additives: uses, exposure, and controversy. Crit Rev Food Sci Nutr 57:3084–3103. 10.1080/10408398.2015.1087964 26587821 10.1080/10408398.2015.1087964

[CR11] Dickey RM, Forti AM, Kunjapur AM (2021) Advances in engineering microbial biosynthesis of aromatic compounds and related compounds. Bioresour Bioprocess 8. 10.1186/s40643-021-00434-x 10.1186/s40643-021-00434-x PMC10992092 38650203

[CR12] Ferrer L, Mindt M, Suarez-Diez M, Jilg T, Zagorščak M, Lee JH, Gruden K, Wendisch VF, Cankar K (2022) Fermentative indole production via bacterial tryptophan synthase alpha subunit and plant indole-3-glycerol phosphate lyase enzymes. J Agric Food Chem 70:5634–5645. 10.1021/ACS.JAFC.2C01042/35500281 10.1021/acs.jafc.2c01042 PMC9100643

[CR13] Ferrer L, Mindt M, Wendisch VF, Cankar K (2023) Indoles and the advances in their biotechnological production for industrial applications. Syst Microbiol Biomanufacturing 4:511–527. 10.1007/S43393-023-00223-X

[CR14] Flachbart LK, Gertzen CGW, Gohlke H, Marienhagen J (2021) Development of a biosensor platform for phenolic compounds using a transition ligand strategy. ACS Synth Biol 10:2002–2014. 10.1021/ACSSYNBIO.1C00165 34369151 10.1021/acssynbio.1c00165

[CR15] Gong Z, Chen J, Jiao X, Gong H, Pan D, Liu L, Zhang Y, Tan T (2024) Genome-scale metabolic network models for industrial microorganisms metabolic engineering: current advances and future prospects. Biotechnol Adv 72:108319. 10.1016/J.BIOTECHADV.2024.108319 38280495 10.1016/j.biotechadv.2024.108319

[CR16] Guo H, Chang S, Jia L, Wang Z, Zhang Q, Zhang G (2021) Advances in the synthesis and applications of raspberry ketone: a review. Flavour Fragr J 36:615–625. 10.1002/FFJ.3678

[CR17] Herrmann KM, Weaver LM (1999) The shikimate pathway. Annu Rev Plant Biol 50:473–503. 10.1146/annurev.arplant.50.1.473 10.1146/annurev.arplant.50.1.473 15012217

[CR18] Hong CS, Jikei M, Kakimoto MA (2003) Synthesis and characteristics of hyperbranched polybenzoxazoles via poly(*o*-hydroxyamide) precursors. Polym J 35:859–867. 10.1295/polymj.35.859

[CR19] Ikeda M (2006) Towards bacterial strains overproducing L-tryptophan and other aromatics by metabolic engineering. Appl Microbiol Biotechnol 69:615–626. 10.1007/S00253-005-0252-Y 16374633 10.1007/s00253-005-0252-y

[CR20] Ito T, Roongsawang N, Shirasaka N, Lu W, Flatt PM, Kasanah N, Miranda C, Mahmud T (2009) Deciphering pactamycin biosynthesis and engineered production of new pactamycin analogues. ChemBioChem 10:2253–2265. 10.1002/CBIC.200900339 19670201 10.1002/cbic.200900339

[CR21] Jackson RJ, Cooper KL, Tappenden P, Rees A, Simpson EL, Read RC, Nicholson KG (2011) Oseltamivir, zanamivir and amantadine in the prevention of influenza: a systematic review. J Infect 62:14–25. 10.1016/J.JINF.2010.10.003 20950645 10.1016/j.jinf.2010.10.003

[CR22] Jiang M, Zhang H (2016) Engineering the shikimate pathway for biosynthesis of molecules with pharmaceutical activities in *E. coli*. Curr Opin Biotechnol 42:1–6. 10.1016/j.copbio.2016.01.016 26921705 10.1016/j.copbio.2016.01.016

[CR23] Jiang Y, Qian F, Yang J, Liu Y, Dong F, Xu C, Sun B, Chen B, Xu X, Li Y, Wang R, Yang S (2017) CRISPR-Cpf1 assisted genome editing of *Corynebacterium glutamicum*. 10.1038/ncomms15179 10.1038/ncomms15179 PMC5418603 28469274

[CR24] Junker N, Poethe SS, Wendisch VF (2025) Two routes for tyrosol production by metabolic engineering of *Corynebacterium glutamicum*. Biotechnol Biofuels Bioprod 18:43. 10.1186/S13068-025-02641-6 40188127 10.1186/s13068-025-02641-6 PMC11971909

[CR25] Kallscheuer N, Kage H, Milke L, Nett M, Marienhagen J (2019a) Microbial synthesis of the type I polyketide 6-methylsalicylate with *Corynebacterium glutamicum*. Appl Microbiol Biotechnol 103:9619–9631. 10.1007/S00253-019-10121-9 31686146 10.1007/s00253-019-10121-9

[CR26] Kallscheuer N, Marienhagen J (2018) *Corynebacterium glutamicum* as platform for the production of hydroxybenzoic acids. Microb Cell Fact 17:70. 10.1186/s12934-018-0923-x 29753327 10.1186/s12934-018-0923-x PMC5948850

[CR27] Kallscheuer N, Menezes R, Foito A, Da Silva MH, Braga A, Dekker W, Sevillano DM, Rosado-Ramos R, Jardim C, Oliveira J, Ferreira P, Rocha I, Silva AR, Sousa M, Allwood JW, Bott M, Faria N, Stewart D, Ottens M, Naesby M, Dos Santos CN, Marienhagen J (2019b) Identification and microbial production of the raspberry phenol salidroside that is active against huntington’s disease. Plant Physiol 179:969–985. 10.1104/pp.18.01074 30397021 10.1104/pp.18.01074 PMC6393794

[CR28] Kallscheuer N, Polen T, Bott M, Marienhagen J (2017a) Reversal of β-oxidative pathways for the microbial production of chemicals and polymer building blocks. Metab Eng 42:33–42. 10.1016/j.ymben.2017.05.004 28550000 10.1016/j.ymben.2017.05.004

[CR29] Kallscheuer N, Vogt M, Bott M, Marienhagen J (2017b) Functional expression of plant-derived *O*-methyltransferase, flavanone 3-hydroxylase, and flavonol synthase in *Corynebacterium glutamicum* for production of pterostilbene, kaempferol, and quercetin. J Biotechnol 258:190–196. 10.1016/J.JBIOTEC.2017.01.006 28143765 10.1016/j.jbiotec.2017.01.006

[CR30] Kallscheuer N, Vogt M, Kappelmann J, Krumbach K, Noack S, Bott M, Marienhagen J (2016a) Identification of the phd gene cluster responsible for phenylpropanoid utilization in *Corynebacterium glutamicum*. Appl Microbiol Biotechnol 100:1871–1881. 10.1007/s00253-015-7165-1 26610800 10.1007/s00253-015-7165-1

[CR31] Kallscheuer N, Vogt M, Marienhagen J (2017c) A novel synthetic pathway enables microbial production of polyphenols independent from the endogenous aromatic amino acid metabolism. ACS Synth Biol 6:410–415. 10.1021/ACSSYNBIO.6B00291 27936616 10.1021/acssynbio.6b00291

[CR32] Kallscheuer N, Vogt M, Stenzel A, Gätgens J, Bott M, Marienhagen J (2016b) Construction of a *Corynebacterium glutamicum* platform strain for the production of stilbenes and (2*S*)-flavanones. Metab Eng 38:47–55. 10.1016/j.ymben.2016.06.003 27288926 10.1016/j.ymben.2016.06.003

[CR33] Kang DH, Ko SC, Heo YB, Lee HJ, Woo HM (2022) RoboMoClo: a robotics-assisted modular cloning framework for multiple gene assembly in biofoundry. ACS Synth Biol 11:1336–1348. 10.1021/ACSSYNBIO.1C00628 35167276 10.1021/acssynbio.1c00628

[CR34] Kawaguchi H, Hasunuma T, Ohnishi Y, Sazuka T, Kondo A, Ogino C (2021) Enhanced production of γ-amino acid 3-amino-4-hydroxybenzoic acid by recombinant *Corynebacterium glutamicum* under oxygen limitation. Microb Cell Fact 20:228. 10.1186/S12934-021-01714-Z 34949178 10.1186/s12934-021-01714-z PMC8697445

[CR35] Kawaguchi H, Sasaki K, Uematsu K, Tsuge Y, Teramura H, Okai N, Nakamura-Tsuruta S, Katsuyama Y, Sugai Y, Ohnishi Y, Hirano K, Sazuka T, Ogino C, Kondo A (2015) 3-Amino-4-hydroxybenzoic acid production from sweet sorghum juice by recombinant *Corynebacterium glutamicum*. Bioresour Technol 198:410–417. 10.1016/J.BIORTECH.2015.09.024 26409852 10.1016/j.biortech.2015.09.024

[CR36] Kim HS, Choi JA, Kim BY, Ferrer L, Choi JM, Wendisch VF, Lee JH (2022) Engineered *Corynebacterium glutamicum* as the platform for the production of aromatic aldehydes. Front Bioeng Biotechnol 10. 10.3389/FBIOE.2022.880277 10.3389/fbioe.2022.880277 PMC9133326 35646884

[CR37] Kinoshita S, Udaka S, Shimono M (1957) Studies on the amino acid fermentation Part I. Production of L-glutamic acid by various microorganisms. J Gen Appl Microbiol 3:193–205. 10.2323/jgam.3.193 15965888

[CR38] Kitade Y, Hashimoto R, Suda M, Hiraga K, Inui M (2018) Production of 4-hydroxybenzoic acid by an aerobic growth-arrested bioprocess using metabolically engineered *Corynebacterium glutamicum*. Appl Environ Microbiol 84:e02587-e2617. 10.1128/AEM.02587-17 29305513 10.1128/AEM.02587-17 PMC5835730

[CR39] Kluczyk A, Popek T, Kiyota T, de Macedo P, Stefanowicz P, Lazar C, Konishi Y (2012) Drug evolution: *p*-aminobenzoic acid as a building block. Curr Med Chem 9:1871–1892. 10.2174/0929867023368872 10.2174/0929867023368872 12369873

[CR40] Kogure T, Inui M (2018) Recent advances in metabolic engineering of *Corynebacterium glutamicum* for bioproduction of value-added aromatic chemicals and natural products. Appl Microbiol Biotechnol 102:8685–8705. 10.1007/s00253-018-9289-6 30109397 10.1007/s00253-018-9289-6

[CR41] Kogure T, Kubota T, Suda M, Hiraga K, Inui M (2016) Metabolic engineering of *Corynebacterium glutamicum* for shikimate overproduction by growth-arrested cell reaction. Metab Eng 38:204–216. 10.1016/J.YMBEN.2016.08.005 27553883 10.1016/j.ymben.2016.08.005

[CR42] Kogure T, Suda M, Hiraga K, Inui M (2021) Protocatechuate overproduction by *Corynebacterium glutamicum* via simultaneous engineering of native and heterologous biosynthetic pathways. Metab Eng 65:232–242. 10.1016/J.YMBEN.2020.11.007 33238211 10.1016/j.ymben.2020.11.007

[CR43] Krömer JO, Nunez-Bernal D, Averesch NJH, Hampe J, Varela J, Varela C (2013) Production of aromatics in *Saccharomyces cerevisiae*-a feasibility study. J Biotechnol 163:184–193. 10.1016/j.jbiotec.2012.04.014 22579724 10.1016/j.jbiotec.2012.04.014

[CR44] Kubota T, Watanabe A, Suda M, Kogure T, Hiraga K, Inui M (2016) Production of para-aminobenzoate by genetically engineered *Corynebacterium glutamicum* and non-biological formation of an *N*-glucosyl byproduct. Orig Res Artic. 10.1016/j.ymben.2016.07.010 27471069 10.1016/j.ymben.2016.07.010

[CR45] Kuepper J, Otto M, Dickler J, Behnken S, Magnus J, Jäger G, Blank LM, Wierckx N (2020) Adaptive laboratory evolution of *Pseudomonas putida* and *Corynebacterium glutamicum* to enhance anthranilate tolerance. Microbiol 166:1025–1037. 10.1099/MIC.0.000982 10.1099/mic.0.000982 33095135

[CR46] Kunjapur AM, Prather KLJ (2015) Microbial engineering for aldehyde synthesis. Appl Environ Microbiol 81:1892–1901. 10.1128/AEM.03319-14 25576610 10.1128/AEM.03319-14 PMC4345389

[CR47] Kurpejović E, Burgardt A, Bastem GM, Junker N, Wendisch VF, Sariyar Akbulut B (2023) Metabolic engineering of *Corynebacterium glutamicum* for L-tyrosine production from glucose and xylose. J Biotechnol 363:8–16. 10.1016/J.JBIOTEC.2022.12.005 36566842 10.1016/j.jbiotec.2022.12.005

[CR48] Kurpejović E, Wendisch VF, Sariyar Akbulut B (2021) Tyrosinase-based production of L-DOPA by *Corynebacterium glutamicum*. Appl Microbiol Biotechnol 105:9103–9111. 10.1007/S00253-021-11681-5 34762142 10.1007/s00253-021-11681-5

[CR49] Labib M, Görtz J, Brüsseler C, Kallscheuer N, Gätgens J, Jupke A, Marienhagen J, Noack S (2021) Metabolic and process engineering for microbial production of protocatechuate with *Corynebacterium glutamicum*. Biotechnol Bioeng 118:4414–4427. 10.1002/BIT.27909 34343343 10.1002/bit.27909

[CR50] Liu Q, Liu Y, Chen Y, Nielsen J (2020) Current state of aromatics production using yeast: achievements and challenges. Curr Opin Biotechnol 65:65–74. 10.1016/j.copbio.2020.01.008 32092624 10.1016/j.copbio.2020.01.008

[CR51] Luo ZW, Cho JS, Lee SY (2019) Microbial production of methyl anthranilate, a grape flavor compound. Proc Natl Acad Sci U S A 166:10749–10756. 10.1073/PNAS.1903875116/10.1073/pnas.1903875116 PMC6561195 31085637

[CR52] Lütke-Eversloh T, Santos CNS, Stephanopoulos G (2007) Perspectives of biotechnological production of L-tyrosine and its applications. Appl Microbiol Biotechnol 77:751–762. 10.1007/S00253-007-1243-Y 17968539 10.1007/s00253-007-1243-y

[CR53] Marchand CH, Salmeron C, Raad RB, Méniche X, Chami M, Masi M, Blanot D, Daffé M, Tropis M, Huc E, Le MP, Decottignies P, Bayan N (2012) Biochemical disclosure of the mycolate outer membrane of *Corynebacterium glutamicum*. J Bacteriol 194:587–597. 10.1128/JB.06138-11 22123248 10.1128/JB.06138-11 PMC3264076

[CR54] Marienhagen J, Bott M (2013) Metabolic engineering of microorganisms for the synthesis of plant natural products. J Biotechnol 163:166–178. 10.1016/j.jbiotec.2012.06.001 22687248 10.1016/j.jbiotec.2012.06.001

[CR55] Marienhagen J, Kennerknecht N, Sahm H, Eggeling L (2005) Functional analysis of all aminotransferase proteins inferred from the genome sequence of *Corynebacterium glutamicum*. J Bacteriol 187:7639–7646. 10.1128/JB.187.22.7639-7646.2005 16267288 10.1128/JB.187.22.7639-7646.2005 PMC1280304

[CR56] Matsuzawa M, Ito J, Danjo K, Fukui K (2024) Vanillin production by *Corynebacterium glutamicum* using heterologous aromatic carboxylic acid reductases. Biotechnol Biofuels Bioprod 17:58. 10.1186/S13068-024-02507-3 38693567 10.1186/s13068-024-02507-3 PMC11064420

[CR57] Milke L, Aschenbrenner J, Marienhagen J, Kallscheuer N (2018) Production of plant-derived polyphenols in microorganisms: current state and perspectives. Appl Microbiol Biotechnol 102:1575–1585. 10.1007/S00253-018-8747-5 29340710 10.1007/s00253-018-8747-5

[CR58] Milke L, Ferreira P, Kallscheuer N, Braga A, Vogt M, Kappelmann J, Oliveira J, Silva AR, Rocha I, Bott M, Noack S, Faria N, Marienhagen J (2019a) Modulation of the central carbon metabolism of *Corynebacterium glutamicum* improves malonyl-CoA availability and increases plant polyphenol synthesis. Biotechnol Bioeng 116:1380–1391. 10.1002/BIT.26939 30684355 10.1002/bit.26939

[CR59] Milke L, Kabuu M, Zschoche R, Gätgens J, Krumbach K, Carlstedt KL, Wurzbacher CE, Balluff S, Beemelmanns C, Jogler C, Marienhagen J, Kallscheuer N (2024) A type III polyketide synthase cluster in the phylum *Planctomycetota* is involved in alkylresorcinol biosynthesis. Appl Microbiol Biotechnol 108:239. 10.1007/S00253-024-13065-X 38407604 10.1007/s00253-024-13065-x PMC10896814

[CR60] Milke L, Kallscheuer N, Kappelmann J, Marienhagen J (2019b) Tailoring *Corynebacterium glutamicum* towards increased malonyl-CoA availability for efficient synthesis of the plant pentaketide noreugenin. Microb Cell Fact 18:71. 10.1186/s12934-019-1117-x 30975146 10.1186/s12934-019-1117-x PMC6460773

[CR61] Milke L, Marienhagen J (2020) Engineering intracellular malonyl-CoA availability in microbial hosts and its impact on polyketide and fatty acid synthesis. Appl Microbiol Biotechnol 104:6057–6065. 10.1007/S00253-020-10643-7 32385515 10.1007/s00253-020-10643-7 PMC7316851

[CR62] Milke L, Mutz M, Marienhagen J (2020) Synthesis of the character impact compound raspberry ketone and additional flavoring phenylbutanoids of biotechnological interest with *Corynebacterium glutamicum*. Microb Cell Fact 19:92. 10.1186/S12934-020-01351-Y 32316987 10.1186/s12934-020-01351-y PMC7175512

[CR63] Mindt M, Beyraghdar Kashkooli A, Suarez-Diez M, Ferrer L, Jilg T, Bosch D, Martins dos Santos V, Wendisch VF, Cankar K (2022) Production of indole by *Corynebacterium glutamicum* microbial cell factories for flavor and fragrance applications. Microb Cell Fact 21:45. 10.1186/S12934-022-01771-Y 35331232 10.1186/s12934-022-01771-y PMC8944080

[CR64] Mindt M, Ferrer L, Bosch D, Cankar K, Wendisch VF (2023) De novo tryptophanase-based indole production by metabolically engineered *Corynebacterium glutamicum*. Appl Microbiol Biotechnol 107:1621. 10.1007/S00253-023-12397-4 36786915 10.1007/s00253-023-12397-4 PMC10006044

[CR65] Mutz M, Brüning V, Brüsseler C, Müller MF, Noack S, Marienhagen J (2024) Metabolic engineering of *Corynebacterium glutamicum* for the production of anthranilate from glucose and xylose. Microb Biotechnol 17:e14388. 10.1111/1751-7915.14388 38206123 10.1111/1751-7915.14388 PMC10832554

[CR66] Mutz M, Kösters D, Wynands B, Wierckx N, Marienhagen J (2023) Microbial synthesis of the plant natural product precursor p-coumaric acid with *Corynebacterium glutamicum*. Microb Cell Fact 22:209. 10.1186/S12934-023-02222-Y 37833813 10.1186/s12934-023-02222-y PMC10576375

[CR67] Nekhaev AI, Maksimov AL (2021) Production of aromatic hydrocarbons from biomass. Pet Chem 61:15–34. 10.1134/S0965544121010023

[CR68] Nešvera J, Pátek M (2011) Tools for genetic manipulations in *Corynebacterium glutamicum* and their applications. Appl Microbiol Biotechnol 90:1641–1654. 10.1007/s00253-011-3272-9 21519933 10.1007/s00253-011-3272-9

[CR69] Nielsen J, Tillegreen CB, Petranovic D (2022) Innovation trends in industrial biotechnology. Trends Biotechnol 40:1160–1172. 10.1016/j.tibtech.2022.03.007 35459568 10.1016/j.tibtech.2022.03.007

[CR70] Noda S, Kondo A (2017) Recent advances in microbial production of aromatic chemicals and derivatives. Trends Biotechnol 35:785–796. 10.1016/j.tibtech.2017.05.006 28645530 10.1016/j.tibtech.2017.05.006

[CR71] Nonaka K, Osamura T, Takahashi F (2023) A 4-hydroxybenzoate 3-hydroxylase mutant enables 4-amino-3-hydroxybenzoic acid production from glucose in *Corynebacterium glutamicum*. Microb Cell Fact 22:168. 10.1186/S12934-023-02179-Y 37644492 10.1186/s12934-023-02179-y PMC10466732

[CR72] Okai N, Miyoshi T, Takeshima Y, Kuwahara H, Ogino C, Kondo A (2016) Production of protocatechuic acid by *Corynebacterium glutamicum* expressing chorismate-pyruvate lyase from E*scherichia coli*. Appl Microbiol Biotechnol 100:135–145. 10.1007/S00253-015-6976-4 26392137 10.1007/s00253-015-6976-4

[CR73] Parise MTD, Parise D, Kato RB, Pauling JK, Tauch A (2020) Azevedo VA de C, Baumbach J (2020) CoryneRegNet 7, the reference database and analysis platform for corynebacterial gene regulatory networks. Sci Data 71(7):1–9. 10.1038/s41597-020-0484-9 10.1038/s41597-020-0484-9 PMC7214426 32393779

[CR74] Poethe SS, Junker N, Meyer F, Wendisch VF (2024) Sustainable production of the drug precursor tyramine by engineered *Corynebacterium glutamicum*. Appl Microbiol Biotechnol 108:499. 10.1007/s00253-024-13319-8 39476177 10.1007/s00253-024-13319-8 PMC11525245

[CR75] Rosch TM, Tenhaef J, Stoltmann T, Redeker T, Kösters D, Hollmann N, Krumbach K, Wiechert W, Bott M, Matamouros S, Marienhagen J, Noack S (2024) AutoBioTech─a versatile biofoundry for automated strain engineering. ACS Synth Biol 13:2227–2237. 10.1021/ACSSYNBIO.4C00298 38975718 10.1021/acssynbio.4c00298 PMC11264319

[CR76] Saeedi M, Khezri K, Seyed Zakaryaei A, Mohammadamini H (2021) A comprehensive review of the therapeutic potential of α-arbutin. Phyther Res 35:4136–4154. 10.1002/ptr.7076 10.1002/ptr.7076 33724594

[CR77] Sato N, Kishida M, Nakano M, Hirata Y, Tanaka T (2020) Metabolic engineering of shikimic acid-producing *Corynebacterium glutamicum* from glucose and cellobiose retaining its phosphotransferase system function and pyruvate kinase activities. Front Bioeng Biotechnol 8. 10.3389/FBIOE.2020.569406 10.3389/fbioe.2020.569406 PMC7511668 33015020

[CR78] Schulz A, Gepp MM, Stracke F, von Briesen H, Neubauer JC, Zimmermann H (2019) Tyramine-conjugated alginate hydrogels as a platform for bioactive scaffolds. J Biomed Mater Res Part A 107:114–121. 10.1002/JBM.A.36538 10.1002/jbm.a.36538 PMC6585978 30256518

[CR79] Schweizer E, Hofmann J (2004) Microbial type I fatty acid synthases (FAS): major players in a network of cellular FAS systems. Microbiol Mol Biol Rev 68:501. 10.1128/MMBR.68.3.501-517.2004 15353567 10.1128/MMBR.68.3.501-517.2004 PMC515254

[CR80] Shende VV, Bauman KD, Moore BS (2024) The shikimate pathway: gateway to metabolic diversity. Nat Prod Rep 41:604–648. 10.1039/d3np00037k 38170905 10.1039/d3np00037k PMC11043010

[CR81] Song J, He Y, Luo C, Feng B, Ran F, Xu H, Ci Z, Xu R, Han L, Zhang D (2020) New progress in the pharmacology of protocatechuic acid: a compound ingested in daily foods and herbs frequently and heavily. Pharmacol Res 161:105109. 10.1016/J.PHRS.2020.105109 32738494 10.1016/j.phrs.2020.105109

[CR82] Suzuki H, Ohnishi Y, Furusho Y, Sakuda S, Horinouchi S (2006) Novel benzene ring biosynthesis from C3 and C4 primary metabolites by two enzymes. J Biol Chem 281:36944–36951. 10.1074/JBC.M608103200 17003031 10.1074/jbc.M608103200

[CR83] Syukur Purwanto H, Kang MS, Ferrer L, Han SS, Lee JY, Kim HS, Lee JH (2018) Rational engineering of the shikimate and related pathways in *Corynebacterium glutamicum* for 4-hydroxybenzoate production. J Biotechnol 282:92–100. 10.1016/J.JBIOTEC.2018.07.016 30031819 10.1016/j.jbiotec.2018.07.016

[CR84] Tachikawa Y, Okuno M, Itoh T, Hirasawa T (2024) Metabolic engineering with adaptive laboratory evolution for phenylalanine production by *Corynebacterium glutamicum*. J Biosci Bioeng 137:344–353. 10.1016/j.jbiosc.2024.01.006 38365536 10.1016/j.jbiosc.2024.01.006

[CR85] Tharmasothirajan A, Melcr J, Linney J, Gensch T, Krumbach K, Ernst KM, Brasnett C, Poggi P, Pitt AR, Goddard AD, Chatgilialoglu A, Marrink SJ, Marienhagen J (2023) Membrane manipulation by free fatty acids improves microbial plant polyphenol synthesis. Nat Commun 14:5619. 10.1038/S41467-023-40947-X 37699874 10.1038/s41467-023-40947-x PMC10497605

[CR86] Tharmasothirajan A, Wellfonder M, Marienhagen J (2021) Microbial polyphenol production in a biphasic process. ACS Sustain Chem Eng 9:17266–17275. 10.1021/ACSSUSCHEMENG.1C05865

[CR87] van Summeren-Wesenhagen PV, Marienhagen J (2013) Putting bugs to the blush: metabolic engineering for phenylpropanoid-derived products in microorganisms. Bioengineered 4(6):355–362. 10.4161/bioe.23885 23851446 10.4161/bioe.23885 PMC3937195

[CR88] Wiklund P, Bergman J (2006) The chemistry of anthranilic acid. Curr Org Synth 3:379–402. 10.2174/157017906777934926

[CR89] Wolf S, Becker J, Tsuge Y, Kawaguchi H, Kondo A, Marienhagen J, Bott M, Wendisch VF, Wittmann C (2021) Advances in metabolic engineering of *Corynebacterium glutamicum* to produce high-value active ingredients for food, feed, human health, and well-being. Essays Biochem 65:197–212. 10.1042/EBC20200134 34096577 10.1042/EBC20200134 PMC8313993

[CR90] Wu X, Liu J, Liu D, Yuwen M, Koffas MAG, Zha J (2022) Biosynthesis of eriodictyol from tyrosine by *Corynebacterium glutamicum*. Microb Cell Fact 21:86. 10.1186/S12934-022-01815-3 35568867 10.1186/s12934-022-01815-3 PMC9107716

[CR91] Xiao S, Wang Z, Wang B, Hou B, Cheng J, Bai T, Zhang Y, Wang W, Yan L, Zhang J (2023) Expanding the application of tryptophan: industrial biomanufacturing of tryptophan derivatives. Front Microbiol 14. 10.3389/FMICB.2023.1099098 10.3389/fmicb.2023.1099098 PMC10076799 37032885

[CR92] Yildiz G, Ronsse F, Van DR, Prins W (2016) Challenges in the design and operation of processes for catalytic fast pyrolysis of woody biomass. Renew Sustain Energy Rev 57:1596–1610. 10.1016/j.rser.2015.12.202

[CR93] Zha J, Zang Y, Mattozzi M, Plassmeier J, Gupta M, Wu X, Clarkson S, Koffas MAG (2018) Metabolic engineering of *Corynebacterium glutamicum* for anthocyanin production. Microb Cell Fact 17:143. 10.1186/S12934-018-0990-Z 30217197 10.1186/s12934-018-0990-z PMC6138892

[CR94] Zha J, Zhao Z, Xiao Z, Eng T, Mukhopadhyay A, Koffas MA, Tang YJ (2023) Biosystem design of *Corynebacterium glutamicum* for bioproduction. Curr Opin Biotechnol 79:102870. 10.1016/j.copbio.2022.102870 36549106 10.1016/j.copbio.2022.102870

[CR95] Zhan C, Lee N, Lan G, Dan Q, Cowan A, Wang Z, Baidoo EEK, Kakumanu R, Luckie B, Kuo RC, McCauley J, Liu Y, Valencia L, Haushalter RW, Keasling JD (2023) Improved polyketide production in *C. glutamicum* by preventing propionate-induced growth inhibition. Nat Metab 57 5:1127–1140. 10.1038/s42255-023-00830-x 10.1038/s42255-023-00830-x 37443355

[CR96] Zhang B, Gou K, Xu K, Li Z, Guo X, Wu X (2024) De novo biosynthesis of β-arbutin in *Corynebacterium glutamicum* via pathway engineering and process optimization. Biotechnol Biofuels Bioprod 17:88. 10.1186/S13068-024-02540-2 38918796 10.1186/s13068-024-02540-2 PMC11197339

[CR97] Zhang C, Zhang J, Kang Z, Du G, Chen J (2015) Rational engineering of multiple module pathways for the production of L-phenylalanine in *Corynebacterium glutamicum*. J Ind Microbiol Biotechnol 42:787–797. 10.1007/S10295-015-1593-X 25665502 10.1007/s10295-015-1593-x

[CR98] Zhu N, Xia W, Wang G, Song Y, Gao X, Liang J, Wang Y (2023) Engineering *Corynebacterium glutamicum* for de novo production of 2-phenylethanol from lignocellulosic biomass hydrolysate. Biotechnol Biofuels Bioprod 16:75. 10.1186/S13068-023-02327-X 37143059 10.1186/s13068-023-02327-x PMC10158149

[CR99] Zhu Z, Chen R, Zhang L (2024) Simple phenylpropanoids: recent advances in biological activities, biosynthetic pathways, and microbial production. Nat Prod Rep 41:6–24. 10.1039/D3NP00012E 37807808 10.1039/d3np00012e

